# Transitions between persistent climate–carbon regimes coincide with elevated Phanerozoic biosphere vulnerability

**DOI:** 10.1038/s41467-026-75655-9

**Published:** 2026-08-04

**Authors:** Ivan Sudakow, Corinne Myers, Andrej Spiridonov, Robertas Stankevič, Georg Feulner, Valerie Livina

**Affiliations:** 1 School of Mathematics and Statistics, The Open University, Milton Keynes, UK; 2 Carl Sagan Center for Research, The SETI Institute, Mountain View, CA USA; 3 Department of Earth and Planetary Sciences, The University of New Mexico, Albuquerque, NM USA; 4 Department of Geology and Mineralogy, Vilnius University, Vilnius, Lithuania; 5 Earth System Analysis, Potsdam Institute for Climate Impact Research (PIK), Member of the Leibniz Association, Potsdam, Germany; 6 Institute of Physics and Astronomy, University of Potsdam, Potsdam, Germany; 7 Department of Data Science & AI, National Physical Laboratory (NPL), Teddington, UK

**Keywords:** Palaeoclimate, Palaeontology, Applied mathematics

## Abstract

Linking abiotic forcing to coherent biotic responses over Phanerozoic time remains difficult to quantify because both environmental drivers and biological dynamics are heterogeneous, irregularly sampled, and affected by proxy and geochronological uncertainty. Here we introduce a regime-based description of the climate–carbon system in which Phanerozoic evolution is organized into a small number of recurring, long-lived mega-climate states separated by relatively sharp transitions. Using recurrence diagnostics on paired carbon and oxygen stable isotope records, together with complementary early-warning indicators, we identify five persistent regimes and their boundaries. We interpret the resulting structure with a conceptual climate–carbon cycle model that admits multiple coexisting equilibria, providing a mechanistic basis for state changes that switch between stable attractors under changing environmental forcings and perturbations. We connect these results to a biosphere vulnerability metric that incorporates biotic turnover with sample-standardized global diversity to show that vulnerability is systematically elevated near major regime transitions. These results suggest that transitions between persistent climate–carbon attractors provide a dynamical context for why some perturbation intervals coincide with heightened biospheric stress, beyond what is expected from more gradual background environmental changes alone.

## Abstract (web summary)

Earth’s climate over the past 539 million years (the Phanerozoic Eon) occupied a few long-lived “mega-climate” regimes. This study shows that life was most vulnerable to extinction during the relatively sharp transitions between these stable states.

## Introduction

The Phanerozoic Earth system (spanning 539 Ma to present) reflects sustained coupling between abiotic processes (e.g., plate tectonics, climate) and the evolution and ecology of life (including biogeochemical cycling). Despite decades of progress, it remains difficult to quantify when and how abiotic variability drives coherent biotic responses, because both the forcing and the biological response are heterogeneous in space and time. Much previous work connects large extinction events to major perturbations of the Earth system^1–4^. Biotic transitions are thus linked to triggers such as large igneous province volcanism or impacts, and to kill mechanisms that include rapid warming and atmospheric CO_2_ increase, ocean anoxia, and acidification^1,2,5–7^. Mass extinctions are therefore a common focus for testing hypotheses of Earth–life dynamics, including extinction selectivity, intensity, recurrence, and recovery patterns that can vary strongly among events^8–11^.

A complementary perspective is that biotic crises may depend not only on the magnitude of perturbations but also on the dynamical organization of the climate–carbon system. Even low-order climate models show that feedbacks can generate multiple equilibria and path dependence under changing forcing^12,13^, motivating the idea that deep-time climate can occupy a small number of persistent regimes separated by relatively sharp transitions. This view aligns with broader work on Earth-system tipping elements, where thresholds and hysteresis emerge from feedback-rich subsystems and where the rate of forcing can influence transitions^14^. On Phanerozoic timescales, geochemical models similarly encode stabilizing feedbacks while allowing excursions when boundary conditions or feedback strengths change^15–17^. Empirically, identifying regimes requires integrating heterogeneous proxy archives: carbonate *δ*^18^O and *δ*^13^C, together with seawater ^87^Sr/^86^Sr, track broad changes in temperature, carbon cycling, and tectonic–weathering balance^18,19^, and multi-proxy *p*CO_2_ syntheses provide radiative-forcing context across the last  ~ 420 Myr^20^. However, deep-time records are irregularly sampled, nonstationary, and affected by geochronological and geo-proxy uncertainties, motivating the use of dynamical systems tools rather than purely correlative comparisons. This large-scale perspective is also important because individual local stratigraphic sections are inevitably incomplete and may be interrupted by hiatuses or unconformities. Accordingly, the present framework is intended for interregional or global composite records, where combining multiple records from different settings helps mitigate local, facies-controlled gaps, rather than for direct application to individual local sections.

Here we combine proxy evidence and dynamical tools to test whether Phanerozoic climate–carbon evolution is best described as residence in a small set of recurring regimes and whether regime transitions align with elevated extinction risk. We compile long time-series of carbon and oxygen stable isotopes^19,21–23^, together with available interpreted paleo-temperature and paleo-atmospheric *p*CO_2_ reconstructions^20,24,25^, and link these to biodiversity histories derived from alternative compilation methods^26,27^. To identify regime structure and coordinated transitions in the coupled carbon–temperature proxy system, we use recurrence diagnostics, including joint-recurrence variants, to quantify changes in dynamical organization through time^28^, and use early warning indicators motivated by critical transition theory^29^ to assess candidate transition intervals.

We then interpret the resulting regime structure with a conceptual climate–carbon cycle model^30^ that admits multiple equilibria and transient perturbations; we quantify biological response using a vulnerability metric that combines turnover with sample-standardized diversity. Together, these analyses support a partition of the Phanerozoic Earth system into a small number of long-lived climate states. We show that transitions between these mega-climate states coincide with elevated extinction and increased macroevolutionary vulnerability. These results are consistent with the hypothesis that climate–carbon perturbations may lead to regime shifts and associated biotic crises.

## Results

### Recurrence-informed mega-climate states

**Fig. 1:**
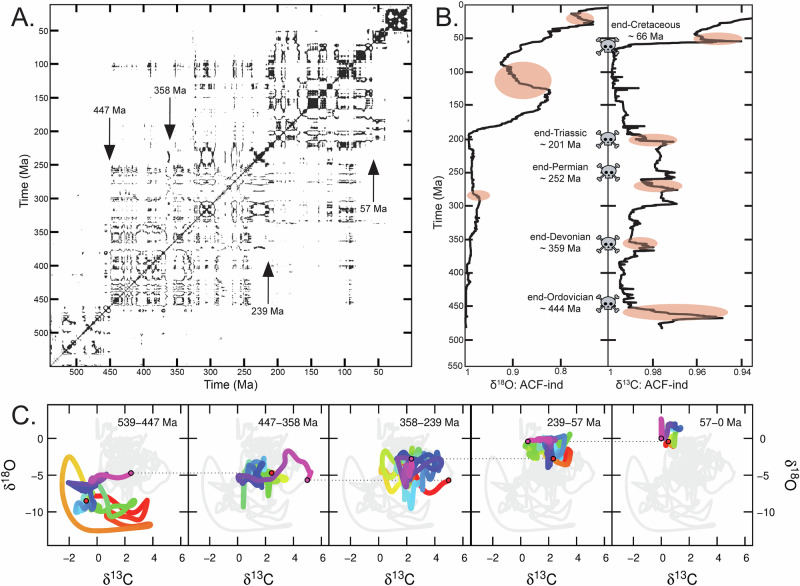
Recurrence-based identification of Phanerozoic mega-climate states and early-warning structure. **A** Joint recurrence plot of *δ*^18^O–*δ*^13^C constructed from the corresponding univariate recurrence plots, using the recurrence-rate settings described in *Methods*. Black arrows mark transitions between Haggis bins (see **C**). **B** Early-warning signal (EWS) analysis of the Phanerozoic *δ*^18^O and *δ*^13^C time series. Red ovals indicate transition points identified by EWS, several of which coincide with transitions between Haggis bins and biospheric events. Gray skulls indicate approximate timing of the “Big 5” mass extinctions. In the *δ*^18^O series, EWS behavior is identified at  ~ 10–30 Ma,  ~ 80–125 Ma, and  ~ 270–280 Ma, potentially related to elevated extinction intervals in the mid-Pliocene, middle Cretaceous, and late Paleozoic, respectively, and associated Earth-system changes (mid-Pliocene warm period, Oceanic Anoxic Event 2, and the late Paleozoic greenhouse transition). In the *δ*^13^C series, EWS behavior is identified at  ~ 30–50 Ma,  ~ 180–200 Ma,  ~ 260–280 Ma,  ~ 350–360 Ma, and  ~ 450–470 Ma. These periods coincide with biotic crises surrounding the Paleocene–Eocene, end-Triassic, late Paleozoic, Serpukhovian, and end-Ordovician intervals, respectively. Associated Earth-system events include the Paleocene–Eocene Thermal Maximum (PETM), the Toarcian Oceanic Anoxic Event (TOAE), Central Atlantic Magmatic Province (CAMP) and Siberian Traps volcanism, transition into the Late Paleozoic Ice Age, and Hirnantian glaciation, respectively. **C** Climate evolution through the Phanerozoic in *δ*^18^O–*δ*^13^C space. Points are colored using an age gradient within each time window, where red is the oldest age in a given bin and magenta is the youngest; shaded background bands indicate the five consolidated mega-climate states used in the main text; endpoints are connected between panels by dotted lines.

**Fig. 2:**
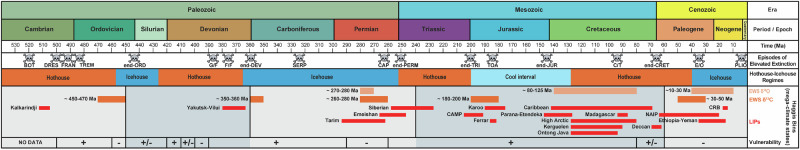
Timeline of relevant geological data and key results. Additional detail is provided in Supplementary Material S6. Gray skulls represent periods of elevated extinction common to^3,26,27^. BOT: late Botomian ( ~ 521–519 Ma); DRES: early Dresbachian ( ~ 501–499 Ma); FRAN: early Franconian ( ~ 497–495 Ma); TREM: late Trempealeauan ( ~ 490–488 Ma); end–ORD: Ordovician–Silurian boundary ( ~ 447–445 Ma); G/F: late Givetian ( ~ 389–385 Ma); F/F: late Frasnian ( ~ 378–374 Ma); end–DEV: Devonian–Carboniferous boundary ( ~ 364–359 Ma); SERP: early Serpukhovian ( ~ 359–326 Ma); CAP: late Capitanian ( ~ 266–260 Ma); end–PERM: Permian–Triassic boundary ( ~ 254–251 Ma); end–TRI: Triassic–Jurassic boundary ( ~ 204–200 Ma); TOA: Pliensbachian–Toarcian boundary ( ~ 186–183 Ma); end–JUR: Jurassic–Cretaceous boundary ( ~ 148–146 Ma); C/T: Cenomanian–Turonian boundary ( ~ 96–94 Ma); end–CRET: Cretaceous–Paleogene boundary ( ~ 68–66 Ma); E/O: Eocene–Oligocene boundary ( ~ 37–34 Ma); PLIO: mid–Pliocene ( ~ 5–2 Ma). Hothouse–Icehouse regimes from^24^. Haggis bins are demarcated by alternating gray and white boxes; EWS analysis results (light and dark orange bars for *δ*^18^O and *δ*^13^C, respectively), LIPs^35^, and calculated biosphere vulnerability is overlaid ("+” indicates v  > 0 suggesting enhanced vulnerability; “–” indicates v  < 0 suggesting decreased vulnerability).

**Fig. 3:**
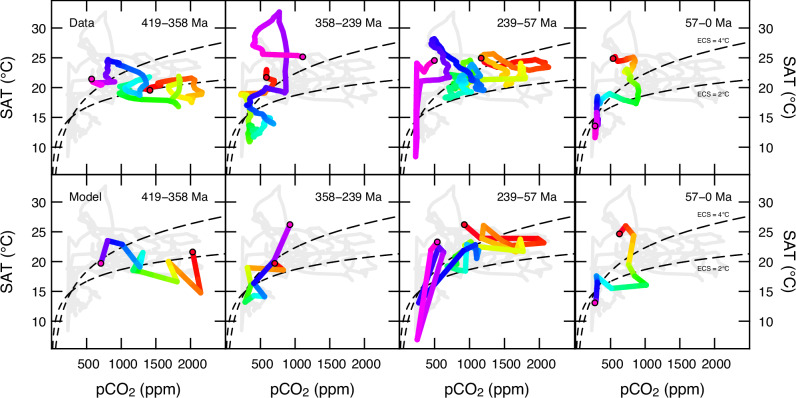
Climate evolution and model response in temperature–*p*CO_2_ space. Upper panels: Climate evolution through the Phanerozoic in temperature–*p*CO_2_ space, shown using the same within-bin age gradient coloring as in Fig. 1C. The oldest mega-climate interval is excluded from this projection because reliable SAT and *p*CO_2_ reconstructions are not available. Lower panels: Response of the conceptual climate–carbon model over the same time intervals; see text for details.

**Fig. 4:**
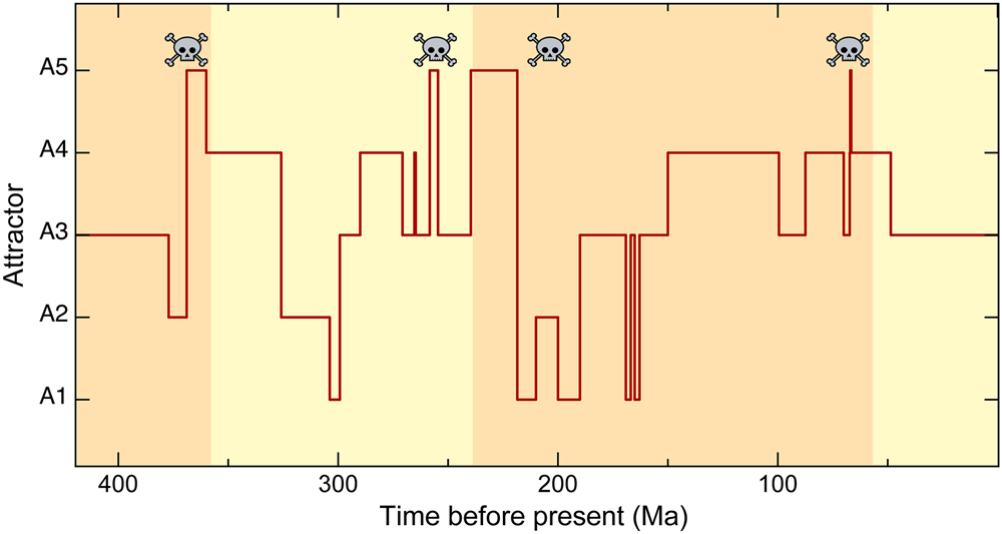
Selected equilibrium branch of the coupled carbon cycle–temperature model through time. Step curve showing the attractor label *A*(*t*) (A1–A5) across the four SAT–*p*CO_2_ mega-climate states used for calibration (shaded bands). At each time point, *A*(*t*) denotes the stable equilibrium branch (root of the energy-balance equation) whose temperature is closest to the reconstructed SAT. Skull symbols mark benchmark biodiversity crisis intervals.

We use recurrence structure in the *δ*^18^O and *δ*^13^C proxy records, together with complementary early-warning indicators, to identify coordinated transitions in climate proxy variables and to segment Phanerozoic proxy trajectories into five long-lived mega-climate states (Fig. 1). Recurrence analysis^28^ (Fig. 1A) results for the Cenozoic Era are similar to the Cenozoic-only analysis by Westerhold et al.^31^, replicating the broad climate behaviors uncovered by these authors (see the youngest time interval in Fig. 1C). Unique from this previous analysis, here we test not only auto-recurrence of the individual climate proxies of *δ*^18^O and *δ*^13^C, but also apply joint recurrence analysis that considers both proxies and their potential interactions. Using these techniques, we identify mega-climate states^32^— henceforth called “Haggis” bins, a descriptive shorthand for the mottled, block-like structure visible in the recurrence plots and inspired by the traditional Scottish dish — across the entire Phanerozoic. These five Haggis bins and their geological and biotic context are summarized in Fig. 2 before being discussed individually below. Additional support for identified transitions between Haggis bins comes from independent analysis of early warning signals^33^ (Fig. 1B) and a conceptual climate–carbon model demonstrating potential mechanics of this process (Figs. 3 and 4).

**Fig. 5:**
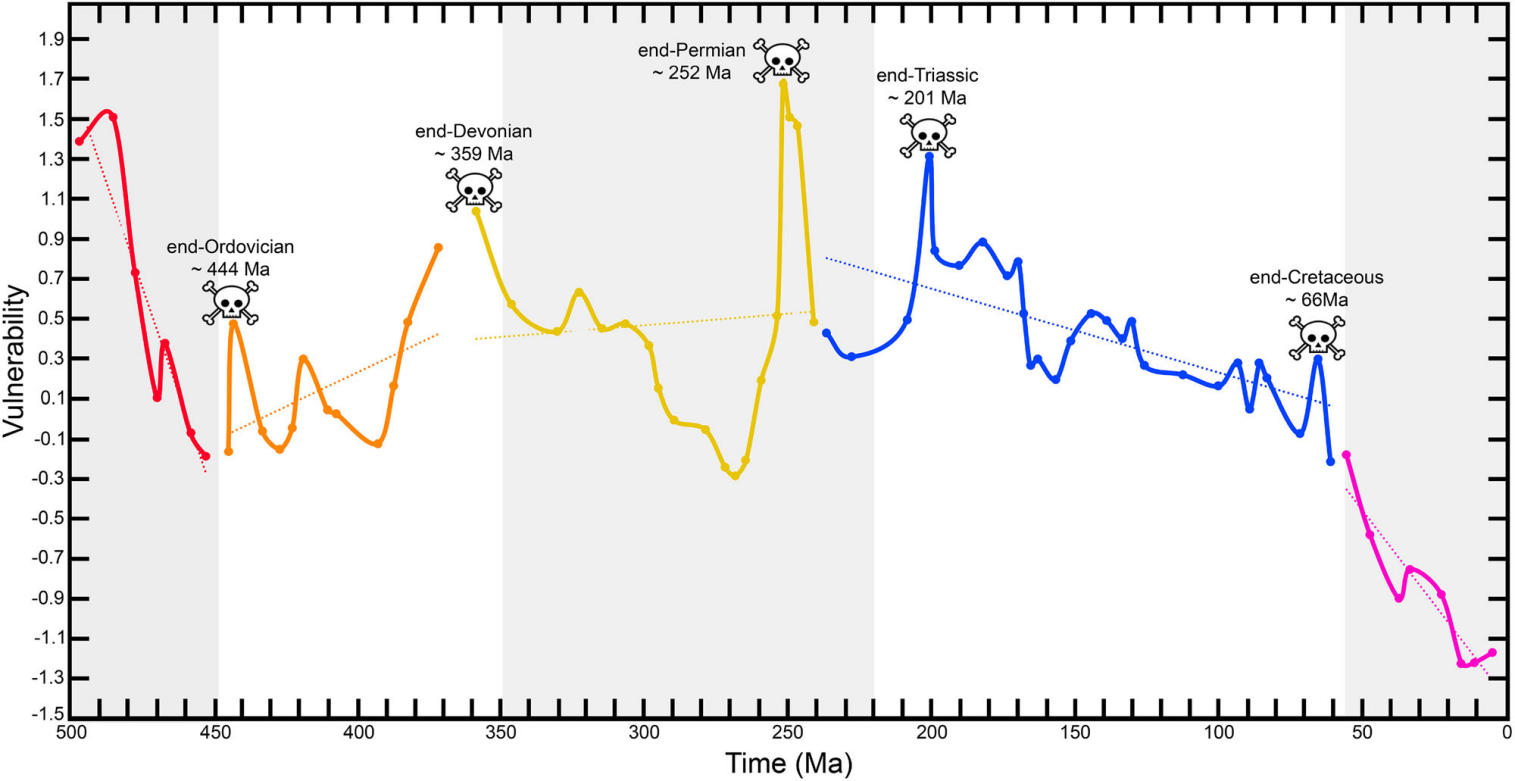
Phanerozoic vulnerability across five mega-climate states. Vulnerability stratified by the five mega-climate states defined in Fig. 1, using color bands ordered from oldest (red) to youngest (magenta). Dotted lines show linear fits within each mega-climate state. Skull symbols mark the widely accepted “Big Five” mass extinctions^8^.

1. **Cambrian–Upper Ordovician** (539–447 Ma). This interval is characterized by the early Paleozoic greenhouse, including the potential transition into the brief Late Ordovician icehouse^24,25^. Both positive and negative carbon isotope excursions (CIEs) are numerous during this period indicating a volatile carbon cycle (e.g.,^34^). The interval also includes the emplacement of the Kalkarindji large igneous province (LIP;  ~ 511–505 Ma;^35^). Taken all together, the environmental regime of this mega-climate state sets the stage for some significant changes in the biosphere, including the Cambrian Explosion (evolutionary origins of most animal phyla around 520 Ma^36^) and the Great Ordovician Biodiversification Event (GOBE;  ~ 485–460 Ma;^37,38^), in which lower taxonomic-level animal diversity increased substantially. Four major periods of elevated global extinction occur across this interval in the late Botomian ( ~ 521–519 Ma), early Dresbachian ( ~ 501–499 Ma), early Franconian ( ~ 497–495 Ma), and late Trempealeauan ( ~ 490–488 Ma)^3,26,27,39^. Early warning analysis (EWS) identifies two *δ*^13^C change points within this interval around 500 Ma, which coincide with the uptick in elevated extinctions (Fig. 1B). This is also reflected in our vulnerability index which is primarily positive over this interval - indicating a global biota that is quite vulnerable to extinction as recognized by others (e.g.,^32^) (Fig. 5).
2. **Upper Ordovician–lowermost Carboniferous** (447–358 Ma). This interval is characterized by the transition out of the end-Ordovician icehouse and into the lower Paleozoic greenhouse^24,25^. The interval includes several, primarily positive, CIEs potentially pointing to distinct periods of substantial organic carbon burial^34,40^. A single, well-dated episode of LIP volcanism, the Yakutsk-Vilui traps ( ~ 380–360 Ma;^35^) is observed. The interval begins with significant biotic upheaval associated with the short-lived end-Ordovician icehouse (Hirnantian glaciation), during which up to 86 percent of the newly evolved biodiversity was lost to mass extinction^41,42^. Through the Silurian and into the Devonian, the biosphere weathered several additional instances of biotic crisis, including the Upper Devonian events at the end-Givetian ( ~ 389–385 Ma), end-Frasnian ( ~ 378–374 Ma), and end-Famenian ( ~ 364–359 Ma) stages, crises associated with negative CIEs and global production of black shales^43,44^. The backdrop for these events includes the rise and establishment of terrestrial biota^45^ and an associated global cooling trend^46,47^, a precursor to the Earth system changes to come in the next Haggis bin. This Haggis bin is bounded on either side by EWS change points in the *δ*^13^C record with variable sign in the vulnerability index throughout the interval (Figs. 1B and 5). This suggests that this interval may represent a minor Earth system quiescence in both carbon cycle regime and biodiversity, bracketed by Earth system disruption. Earth system disruption associated with Haggis bin transitions is further demonstrated by an independent high-resolution plankton diversity record (Graptoloidea), which exhibits a punctuated, two-episode EWS structure across the Late Ordovician (see Supplementary Material S5). This illustrates the multi-scale nature of Haggis-bin transitions—reflected in both the fossil and geochemical records—and their impacts on the biosphere.
3. **Carboniferous–Middle Triassic** (358–239 Ma). This interval spans the transition into and out of the late Paleozoic icehouse^24,25,48,49^ as well as the final assembly of the Pangaean supercontinent. Several negative CIEs are observed in this interval, some of very large magnitude that reflect significant Earth system events (e.g., the Permo-Triassic transition  ~ 252 Ma;^34^). Leading into the interval, a positive CIE is linked to organic carbon burial associated with the terrestrial revolution and formation of large fossil fuel deposits. Later CIEs are associated with the emplacement of three, well-dated LIPs (Tarim  ~ 292–262 Ma, Emeishan  ~ 265–247 Ma, and Siberian Traps  ~ 257–228 Ma;^35^). The Siberian Traps in particular mark significant Earth system change, including both the emergence of the Mesozoic Greenhouse, a period of enhanced climate variability^50^, and the largest mass extinction event in Phanerozoic history, where up to  ~ 95 percent of species went extinct^3,51,52^. Three smaller extinction events also characterize this interval in the Serpukhovian ( ~ 359–326 Ma;^53^), Capitanian ( ~ 266–260 Ma;^54^), and Smithian-Spathian ( ~ 250 Ma;^55,56^). The first of these is associated with an EWS in the *δ*^13^C record, whereas the latter two are preceded by an EWS signal in both the *δ*^13^C and *δ*^18^O records (Fig. 1B). Notably, although not demonstrating the largest decrease in biodiversity, the Serpukhovian event is characterized by significant ecological upheaval^53^ and decrease in carbonate production^57^. Unsurprisingly, this time of upheaval is primarily characterized by positive vulnerability scores, supporting a biosphere potentially predisposed for elevated extinction (Fig. 5).
4. **Middle Triassic–early Paleogene** (239–57 Ma). This interval spans the Mesozoic greenhouse, including a short-lived cooling in the Jurassic^24,25,58^, and breakup of the Pangaean supercontinent^59^. This lengthy interval is characterized by a highly volatile carbon cycle, demonstrated by the many positive and negative CIEs observed. The interval experienced the most LIP volcanism of any interval, including the completion of the Siberian Traps, and emplacement of the CAMP ( ~ 204–191 Ma), Karoo-Ferrar ( ~ 189–175 Ma), Paraná–Etendeka ( ~ 146–127 Ma), Ontong Java ( ~ 127–93 Ma), Kerguelen ( ~ 127–90 Ma), High Arctic ( ~ 127–80 Ma), Madagascar ( ~ 93–88 Ma), Caribbean ( ~ 142–69 Ma), Deccan Traps ( ~ 69–64 Ma), and North Atlantic Igneous Province (NAIP;  ~ 64–20 Ma) events^35,60^. The biosphere also experienced significant upheaval during this period including six elevated extinction events (middle-Triassic  ~ 232 Ma; end-Triassic  ~ 204–200 Ma; early-Jurassic  ~ 186–183 Ma; late-Jurassic  ~ 148–145 Ma; mid-Cretaceous  ~ 95–94 Ma; and end-Cretaceous  ~ 68–66 Ma,^3,26,27,60–66^). Extinction rates were variable, but the end-Triassic and end-Cretaceous stand out with species losses between 75–85%^3,26,61,62^. These two events are also characterized by a cooling-warming sequence in the wake of the CAMP eruptions^67^ and a marked cooling^68^ as well as a marine productivity spike^69^ due to the Chicxulub asteroid impact, respectively. Concurrently, this interval reflects two additional biospheric events: the Mesozoic Marine Revolution^70,71^, wherein radiation of marine phytoplankton resulted in ecological innovation up the food chain and a substantial shift in the carbon buffering capacity of the oceans^72^; and the evolution and expansion of angiosperm plants on land^73^. Given the degree and frequency of environmental change, it is unsurprising that the vulnerability index begins positive, however it decreases until becoming negative by the end of the interval (Fig. 5). Early warning signal analyses detect a single change-point  ~ 200 Ma in the *δ*^13^C record, which is associated with the transition from the previous Haggis bin as well as preceding the end-Triassic and Toarcian elevated extinctions. A single EWS change point was also detected in the *δ*^18^O record, which precedes the end-Cretaceous elevated extinction event (Fig. 1B).
5. **Post-Paleocene–Cenozoic** (57–0 Ma). This interval is characterized by the transition from the Mesozoic greenhouse into the Cenozoic icehouse^24,25,31^, including severe glacial-interglacial temperature swings of the Plio-Pleistocene, the isolation of Antarctica, the collision of India with Asia, and closure of the Tethys Sea by formation of the Isthmus of Panama as the Earth’s tectonic plates reached their modern positions^74,75^. Several positive CIEs are observed^34^ spanning the emplacement of three main LIP events (NAIP  ~ 64–44 Ma, Ethiopia-Yemen  ~ 34–16 Ma, and Columbia River Basalts  ~ 17–6 Ma;^35^). The interval includes two elevated extinction events at the Eocene-Oligocene ( ~ 37–34 Ma) and mid-Pliocene ( ~ 5–2 Ma), however extinction rates at both of these events are quite low in comparison to other intervals^3,26,27,76^. The vulnerability index is negative and declining throughout this interval, reflecting the extinction resistance apparent in the biosphere at this time (Fig. 5). Early warning signal analyses identify two change points, one  ~ 50 Ma in the *δ*^13^C record and the other  ~ 20 Ma in the *δ*^18^O record (Fig. 1B). The former EWS precedes the Eocene climate optima and Eocene-Oligocene extinction; the latter is associated with onset of the main phase of Columbia River Basalt emplacement and mid-Pliocene extinction^31^.

The Haggis bins described here provide a concise characterization of recurrent elements in isotope phase space that can be compared with changes in the Earth system and macroevolutionary dynamics built from origination, extinction, and standing diversity. Across the corresponding intervals we recover familiar Earth system reorganizations, including mass extinctions, large igneous province (LIP) emplacement, carbon isotope excursions, among others (see Supplementary Material S6).

Projecting the same time axis into the calculated proxies of SAT (global surface air temperature)–*p*CO_2_ space (derived from^20,24^, respectively) shows that each Haggis bin occupies a distinct region of the empirical temperature–*p*CO_2_ manifold rather than drifting smoothly along a single branch (Fig. 3). Note that only four Haggis bins are reconstructed here, because *p*CO_2_ data records exist only for the past  ~ 420 Myr^20^. The observed trajectories form curved bands consistent with a SAT response to *p*CO_2_, but the system typically resides for tens of Myr on one portion of the manifold before shifting to another (for example, a Late Paleozoic icehouse or a Mesozoic greenhouse). This could imply that climate sensitivity, a measure of the warming response of the Earth system to *p*CO_2_ increase, varies as a function of long-term background (mega-climate) states.

### Dynamical interpretation from a conceptual climate–carbon model

To interpret the SAT–*p*CO_2_ manifold dynamically, we calibrated a conceptual climate–carbon cycle model^30^ in the four Haggis bins that include *p*CO_2_ data. The model includes an excitable carbon cycle with temperature-dependent weathering and impulsive inputs (e.g., carbon injection from LIPs or asteroid impacts) that is coupled to a simple energy balance with ice–albedo feedbacks and logarithmic greenhouse forcing (see *Methods*). The calibrated model reconstructs 1-Myr quasi-equilibrium trajectories in SAT–*p*CO_2_ space (Fig. 3). The trajectories preserve temporal ordering along each curve and follow the expected SAT response to *p*CO_2_, indicating that the model reproduces the *joint* SAT–*p*CO_2_ structure rather than simulating temperature alone.

Because the equilibrium condition can admit multiple coexisting solutions, the same reconstruction yields a discrete attractor (equilibrium-branch) label *A*(*t*) ∈ {A1, …, A5} (Fig. 4) that identifies which solution is selected at each time. In contrast to proxy-based regime classifications (e.g., temperature categories such as “icehouse/greenhouse”^24,25^), *A*(*t*) does not delineate temperature, but instead the dynamical complexity of the climate–carbon cycle system. Multi-Myr plateaus shown in Fig. 4 indicate sustained residence on one branch, whereas step-like changes reflect switching between branches and/or reorganization of the equilibrium landscape under transient forcing. Short-lived notches correspond to brief excursions, whereas more sustained reorganizations align with major perturbations (e.g., biospheric intervals such as the “Big 5” mass extinctions).

### Macroevolutionary vulnerability across climate states

Macroevolutionary stress refers to the suite of factors that result in global biodiversity decline, change to abundances and ecological structure, and extinction^77^. We quantify macroevolutionary stress using a vulnerability metric derived from standing generic diversity and turnover rates (see *Methods*;^26,27^). Here turnover is defined as the sum of origination and extinction rates, *p* + *q*, and is used as a measure of the overall intensity of biotic reorganization. It captures how rapidly taxa enter and leave the standing biota, rather than only the net balance between origination and extinction. Thus, elevated turnover indicates rapid restructuring of the standing biota, whether through loss, replacement, or recovery. Vulnerability  > 0 indicates a “stressed” global biosphere, whereas vulnerability  < 0 indicates a less stressed and therefore potentially less vulnerable biosphere. Over the Phanerozoic vulnerability behaves nonlinearly and displays several pronounced peaks in the early and mid-Paleozoic and around the Permian–Triassic transition, followed by a sustained decline from the Early Triassic into the late Cenozoic (Fig. 5). This late decline may reflect the combination of overall increasing diversity, damping of extinction, and accumulation of longer-lived taxa^8,78–80^. Notably, periods of elevated Phanerozoic extinction (including the “Big 5” mass extinctions;^3^) are associated with vulnerability peaks. All but the smallest interval (mid-Pliocene,  ~ 1.8–5.3 Ma) of elevated extinction identified by several authors^3,26,27^ are associated with vulnerability scores  > 0. This suggests that biospheric stress, in concert with climate forcing or perturbation, may tip the global biota into mass extinction. Importantly, this non-dimensional vulnerability metric is agnostic to method of diversity estimation. We compared two diversity, extinction, and origination methods: the commonly applied three-timer (3T) estimation protocol of^26^ and the per-capita (PC) estimation of^27^. Both methods provide congruent vulnerability patterns; see Supplementary Material S1.2 and S2.3 for details.

At the scale of the full Phanerozoic, vulnerability is positively associated with the *δ*^18^O temperature proxy (Supplementary Material S2.3). However, within individual Haggis bins the *δ*^18^O–vulnerability relationship, although frequently positive, is more variable in slope (Fig. 5, dotted lines). We interpret this to suggest that background biotic stress is potentially conditioned primarily by long-time-scale warm mega-climate background states, while shorter epoch- and stage-scale conditions can be associated with a wide range of vulnerability levels.

Stratifying vulnerability by the five Haggis bins shows systematic contrasts between mega-climate states (Fig. 5). Two long intervals stand out: the Cambrian greenhouse shows consistently high vulnerability (comparable to greenhouse P–Tr and early Triassic vulnerability values), whereas the overall cool Cenozoic has the lowest vulnerability. This contrast is consistent with positive scaling of mega-climate variability^32,81^ and its influence on biodiversity, which implies that the largest background temperature differences emerge on the longest time scales, placing the Cambrian and Cenozoic at opposite Phanerozoic extremes. Together, these results suggest that long timescale thresholds in global surface temperatures could contribute to changes in background extinction risk, turnover, and global biodiversity levels^82^.

These observations are also consistent with extremely high volatility in the early Paleozoic and low macroevolutionary volatility in the Cenozoic identified previously^83^. Volatility is defined by these authors as the sum of extinction and origination divided by standing biodiversity (similar to the vulnerability index used here, but without diversity standardization and on an arithmetic rather than logarithmic scale). Declining volatility from the middle Mesozoic onwards may be related to long-term biological sorting (as suggested in Ref. ^83^); however, the strong correlation between Phanerozoic temperature and vulnerability ( ~ volatility) would suggest an important contribution of long-term climatic state in setting the background regime of biospheric stress and its effects on global biodiversity.

## Discussion

Our results suggest that Phanerozoic climate (comprising the past 539 million years of Earth history) did not wander randomly through an unstructured series of states, but instead spent most of its time bounded within a small set of recurring regimes. Strikingly, these regimes emerge consistently when we view the system in different ways: joint recurrence structure in paired *δ*^18^O–*δ*^13^C analyses, early-warning indicators, and conceptual climate–carbon cycle modeling in SAT–*p*CO_2_ space all partition the last  ~540 Myr into a similar sequence of long-lived climate states separated by relatively sharp transitions (Supplementary Material S6). The convergence across diagnostics is important: it implies that the bins are not artifacts of any particular proxy compilation or statistical method, but reflect something robust about the climate system. Thus, the Haggis bins described here provide a concise perspective on recurrent pieces of climate–carbon isotope phase space and help to direct further investigation into transitions between bins, as well as the structural stabilities supporting them.

In light of the behavior of SAT-*p*CO_2_ across the four most recent Haggis bins (Fig. 3), we applied a conceptual climate–carbon cycle model to support a dynamical interpretation of this phenomenon that closely follows the observed bands in each Haggis bin. Because the equilibrium condition can admit multiple coexisting solutions, the model naturally produces quasi-stable equilibrium states separated by bifurcations. In this setting, modest sustained changes in background forcing can reshape the equilibrium landscape such that finite perturbations (e.g., LIP pulses) trigger switching between available equilibrium solutions.

Building on this dynamical interpretation, we next ask what a regime-based organization of climate–carbon cycle phase space implies for long-term biospheric change, and for how macroevolutionary stress should be analyzed in deep time. There is enduring debate over what drives Phanerozoic biodiversity change: some explanations emphasize intrinsically generated dynamics (e.g., self-regulation, escalation, competition), whereas others emphasize extrinsic forcing through large-scale abiotic change^10,84^. In the latter view, climate is only one component of a broader shift in boundary conditions that can also involve changes in ocean buffering capacity and tectonic regime^85^. Our contribution is not to choose among these drivers, but to characterize a compact set of recurring climate–carbon cycle backgrounds within which the relative roles of these factors can be tested.

From this state-conditioned perspective, vulnerability is not well captured by a single stationary relationship with environments across deep time. Across the whole Phanerozoic vulnerability shows a positive association with temperature proxies, yet within individual Haggis bins the temperature–vulnerability relationship is more heterogeneous, with variable slopes and substantial within-regime scatter. The contrast is clearest between persistently elevated vulnerability during early Phanerozoic greenhouse intervals and the low vulnerability of the overall cool late Cenozoic, while boundary intervals show systematically higher vulnerability. Together, these patterns support a hierarchical picture in which long-lived regimes set the baseline stress landscape, and shorter-time environments and event-scale perturbations explore that landscape without necessarily requiring a regime change^86^.

Mechanistically, reduced SAT–*p*CO_2_ models that admit multiple coexisting equilibria make this structure plausible: modest sustained shifts in forcing can reshape the equilibrium landscape and increase susceptibility to finite perturbations, so that regime boundaries become natural candidate windows for heightened biospheric stress^9^. This does not imply that every extinction pulse is “caused” by a state transition, but it motivates sharper questions: which feedbacks provide the structural glue that stabilizes each regime for tens of millions of years, and which combinations of slow forcing and discrete perturbations most readily promote switching between equilibria?

Several limitations remain, including the appropriate time-scale separation for early-warning indicators in deep time, the heterogeneous relationship between Haggis boundaries and different classes of extinction events, the fact that the present framework is designed for interregional or global composite records rather than individual local sections, where local hiatuses, unconformities, or facies-controlled gaps may mimic synchronous change across multiple proxies, and the need for modeling approaches that separate background-regime effects from short-term stressors; we discuss these in detail in Supplementary Material S4. Regardless, the Haggis bins identified here offer a practical “coordinate system” for linking proxy dynamics, conceptual modeling, and macroevolutionary stress. A direct next step is to embrace state-conditioning explicitly: (i) in models, apply comparable short-term stressors to different calibrated background equilibria to test whether regime-dependent sensitivity yields systematically different extinction sized responses and recoveries; and (ii) in data, replicate the regime-stratified vulnerability analyses across specific clades, functional groups, regions, and temporal resolutions to identify which signals are robust and which are contingent. In doing so, the Haggis bin framework can help separate baseline stress from event triggers and generate testable predictions about when and why the biosphere becomes most vulnerable.

## Methods

### Data and data processing

#### Environmental data

Environmental data describing Phanerozoic temperature and carbon-cycle variability—including raw *δ*^18^O and *δ*^13^C time series (compiled from^21^), and reconstructions of SAT (global surface air temperature;^24^) and atmospheric *p*CO_2_^20^ where available—were used for recurrence-based regime diagnostics and for projections into SAT–*p*CO_2_ space. Analyses in SAT–*p*CO_2_ space were restricted to intervals where both SAT and *p*CO_2_ reconstructions are available and meet the inclusion criteria (i.e., 0–420 Ma).

All time series were pre-processed to enable recurrence-based diagnostics and comparable phase-space projections. Because recurrence diagnostics and AR(1)-based early-warning indicators are most interpretable on temporally comparable, uniformly sampled series and can be sensitive to high-frequency noise fluctuations, we pre-processed the isotope proxy series by (i) interpolating each series onto an equidistant time grid while keeping the same number of samples, and (ii) denoising each series using a 1D Gaussian filter. The raw *δ*^13^C and *δ*^18^O compilations used here differ substantially in sampling density and age spacing; accordingly, this preprocessing was introduced so that the same window-based and recurrence-based analyses could be applied consistently to both series while avoiding oversampling that could introduce artificial autocorrelation. For SAT–*p*CO_2_ phase-space plots, which require pointwise correspondence between series, we interpolated the coarser-resolution series onto the grid of the finer-resolution series to match sample counts. All interpolation and filtering steps were performed using built-in routines in MathWorks MATLAB (R2022b).

For recurrence-based diagnostics and EWS estimation on *δ*^13^C and *δ*^18^O, we additionally resampled both isotope series to a common fixed uniform grid at 0.472 Myr resolution from 538.5 Myr to 0.018 Myr (*N* = 1140). This equal-spacing step applies to the isotope-series recurrence and EWS analyses only. See Supplementary Material S1.1 for additional information regarding parametrization of these analyses.

#### Macroevolutionary data

Macroevolutionary dynamics are commonly summarized by standing diversity (*D*), origination rates (*p*), and extinction rates (*q*), with derived quantities such as the net diversification rate (*r* = *p* − *q*) (e.g.,^26,79^). We analyzed global marine invertebrate *D*, *p*, and *q* time series using two complementary datasets derived from biodiversity data in the Paleobiology Database (paleodb.org): Alroy’s three-timer (3T) framework (mid-point ages; 516.5–3.62 Ma, 53 points;^26^) and Foote’s per-capita (PC) framework (ages-at-base; 497–5.3 Ma, 74 points;^27^). Because the PC diversity time series was logged, it was back-transformed before vulnerability analysis to the arithmetic scale.

Because different estimation frameworks can be more or less conservative in how biodiversity data are retained or filtered out of the analysis, we perform all analyses using both reconstructions on their native time grid and then harmonize them by interpolating the 3T-based vulnerability to the PC-based vulnerability age grid and estimating a near-constant vertical offset. Agreement statistics and alignment variants are reported in Supplementary Material S1.2.

We focus on genus-level rates (rather than species-level) because genus-level fossil records are generally better sampled and less sensitive to sampling/preservation biases. Genera also tend to have stratigraphic ranges that are resolvable within the stage-scale  ~ 10 Myr bins used here. This makes genus-level estimates of diversity, origination, and extinction more consistent with the temporal resolution of the environmental proxy data than species-level estimates.

#### Vulnerability index

The vulnerability metric combines total turnover, *p*_*i*_ + *q*_*i*_, with standardized standing diversity, ${D}_{i}/\overline{D}$, to quantify biotic change considering both origination and extinction^76,87^:

1 $${V}_{i}=\ln \left(\frac{{p}_{i}+{q}_{i}}{{D}_{i}/\bar{D}}\right).$$

where *D*_*i*_ is standing generic diversity in bin *i* and $\bar{D}$ is the mean standing generic diversity. Here *p*_*i*_ + *q*_*i*_ is interpreted as a measure of total turnover intensity rather than net diversification; *D*_*i*_ denotes standing generic diversity and does not explicitly include functional diversity or functional redundancy. This definition yields higher vulnerability when turnover is elevated relative to diversity, and lower vulnerability when turnover is modest and diversity is high^8,79,80,88^. We use the logarithm to stabilize scale, compress the dynamic range, and express vulnerability as an additive contrast between turnover and diversity.

### Early warning signal indicators

We quantified early warning signals (EWS) of critical slowing down using lag-1 autocorrelation (AR(1)), a standard measure of short-term persistence in a time series. AR(1) is expected to increase as recovery from perturbations slows near a critical transition^89,90^. Prior to EWS indicator estimation, each series was detrended to isolate fluctuation dynamics around the long-term trajectory. We computed lag-1 autocorrelation within a 20 Myr sliding window^91^. Indicator values were assigned to the end of each window.

Because pre-processing can shift absolute autocorrelation baselines, we focused on relative temporal trends in the indicator and quantified monotonic changes using Kendall rank correlation. Robustness was assessed via empirical sensitivity analyses in which we varied the sliding-window length and detrending settings (Supplementary Material S1.1); the resulting spread across runs defines uncertainty bounds for the average indicator curves. Early warning signal patterns were interpreted alongside joint recurrence diagnostics to delineate candidate transition intervals (Fig. 1B)^14,92^.

### Recurrence diagnostics

#### Recurrence structure

We quantified coordinated structure and transitions in paired global marine stable isotope time series, *δ*^13^C and *δ*^18^O, using univariate recurrence plots (RPs), joint recurrence plots (JRPs), and a bivariate autorecurrence variant^28,31,93,94^. RPs were constructed without time-delay embedding on the smoothed isotope series, with thresholds selected to enforce fixed recurrence rates (RR  = 20% for univariate RPs; RR  = 10% for JRPs and bivariate autorecurrence). JRPs were formed as the element-wise (Hadamard) product of the two univariate recurrence matrices, retaining only coincident recurrences.

#### Bin boundaries and consolidation

Block-like textures along the line of identity indicate quasi-stationary intervals in which isotope states recur over multi-Myr spans, whereas abrupt changes in texture or extended low-recurrence regions indicate transitions. Because JRPs retain only coincident recurrences, coordinated boundaries appear sharper when both proxies change together; we used these coordinated changes (in conjunction with EWS trends) to delimit candidate climate–carbon regime boundaries, with the finer partitioning and additional diagnostics reported in Supplementary Material S2.1 and S2.2.

### Conceptual climate–carbon cycle model

We use a reduced global climate–carbon cycle model^30^ to interpret the geometry of the empirical SAT–*p*CO_2_ trajectories. The model couples (i) a Budyko–Sellers-type energy-balance framework with temperature-dependent ice–albedo feedback and logarithmic greenhouse forcing, and (ii) a simple ocean–atmosphere carbon-cycle balance in which CO_2_ sources are opposed by temperature-dependent silicate weathering. On Myr time scales, we treat the climate as quasi-equilibrated relative to carbon-cycle adjustment, and therefore reconstruct SAT from an equilibrium constraint rather than by integrating a fast temperature-tendency equation.

At each time step we solve for global surface air temperature, *T*, given reconstructed atmospheric *p*CO_2_ concentration, *C*, by imposing the steady energy-balance condition

2 $$0=1-A(T;{T}_{{{{\rm{alb}}}}})+\varepsilon {\left(\frac{T}{{T}_{0}}\right)}^{4}+b\log \left(\frac{C}{{C}_{0}}\right).$$

where *A*(*T*; *T*_alb_) is a smooth sigmoidal albedo function (set by the characteristic transition temperature *T*_alb_), *T*_0_ and *C*_0_ are reference values, *ε* sets effective long-wave emission, and *b* controls the SAT response to log(C).

Because the albedo feedback makes the equilibrium condition nonlinear, for fixed *C* the equilibrium constraint can admit multiple solutions *T*, corresponding to alternative equilibrium branches (bistability due to ice–albedo feedback). We therefore identify all coexisting solutions by scanning in *T* and detecting zeros of the equilibrium residual, yielding up to five candidate equilibria at a given time. We label these solutions A1–A5 and construct a discrete attractor record *A*(*t*) ∈ {A1, …, A5} by selecting, at each time step, the equilibrium whose temperature is closest to the reconstructed paleotemperature for that time. This procedure yields both (i) a continuous reconstructed trajectory in SAT–*p*CO_2_ space (Fig. 3), and (ii) a stepwise branch-occupancy time series that summarizes long plateaus (persistent branch residence) and abrupt switches (branch transitions or reorganization of the equilibrium landscape) (Fig. 4).

Carbon-cycle forcing is represented as an excitable carbon cycle with a baseline source balanced by a temperature-dependent silicate-weathering sink, and with impulsive inputs (LIPs/impacts) represented as pulses in the source term (positive for LIP-style CO_2_ injections and negative for impact-style draw-downs). These impulsive inputs allow transient excursions that can trigger switching between coexisting equilibria when the system lies near a bifurcation.

Model parameters are calibrated independently within four broad time windows (Haggis bins) defined by the recurrence-based partition of the Phanerozoic interval where both SAT and *p*CO_2_ reconstructions are available. Parameters are estimated by maximum-likelihood fitting assuming independent Gaussian errors in the discrete-time carbon-cycle formulation. Full details of the estimation procedure are provided in Supplementary Material S3.

## Supplementary information

Supplementary Information

Peer Review file

## Data Availability

The source data underlying the main and Supplementary Figs., together with the raw and numerical data used in the analyses, are available in Zenodo at 10.5281/zenodo.20181322. Source data are provided with this paper. Publicly available source datasets used in this study are cited in the manuscript and documented in the deposited source data files.

## Code availability

The code used in this study is available on Zenodo at 10.5281/zenodo.20181322. The analyses were performed in MATLAB R2022b (MathWorks, Natick, MA, USA) and Yorick (https://github.com/llnl/yorick) using standard numerical routines and scripts included in the code repository; the recurrence diagnostics were calculated using custom Perl scripts included in the deposited code package.
