# Supplementary material for: Transitions between persistent climate–carbon regimes coincide with elevated Phanerozoic biosphere vulnerability
Source: Nat Commun. 2026 Aug 4;17:7559. doi: 10.1038/s41467-026-75655-9 (PMC13439174; doi:10.1038/s41467-026-75655-9)
Supplement: Supplementary file 1 [file 41467_2026_75655_MOESM1_ESM.pdf]

Supplementary Material for:
Transitions between persistent climate–carbon regimes coincide with elevated Phanerozoic biosphere vulnerability

Ivan Sudakow[1,2,*], Corinne Myers[3], Andrej Spiridonov[4], Robertas Stankevič[4], Georg Feulner[5,6], Valerie Livina[7]

[1]School of Mathematics and Statistics, The Open University, Milton Keynes, United Kingdom
[2]Carl Sagan Center for Research, The SETI Institute, Mountain View, California, USA
[3]Department of Earth and Planetary Sciences, The University of New Mexico, Albuquerque, New Mexico, USA
[4]Department of Geology and Mineralogy, Vilnius University, Vilnius, Lithuania
[5]Earth System Analysis, Potsdam Institute for Climate Impact Research (PIK), Member of the Leibniz Association, Potsdam, Germany
[6]Institute of Physics and Astronomy, University of Potsdam, Potsdam, Germany
[7]Department of Data Science, National Physical Laboratory (NPL), Teddington, United Kingdom

*Correspondence: `ivan.sudakow@open.ac.uk`

S1 Data processing

S1.1 Data pre-processing and EWS parameter selection.

Paleoclimate proxy series are irregularly sampled and contain gaps and chronological uncertainties; therefore, EWS estimation required pre-processing to achieve temporally equidistant sampling and to reduce high-frequency noise.

Equidistant sampling and filtering. As detailed in the Main Text, proxy time series were interpolated to a fixed 0.472 Myr grid and smoothed using a 1D Gaussian filter. These steps reduce the sensitivity of AR(1) estimation to irregular sampling and high-frequency fluctuations.

Sliding-window length. EWS indicators were computed in sliding windows. A conventional choice (half the record length) would average over hundreds of Myr for Phanerozoic-scale data and would obscure epoch- and stage-scale reorganizations. We therefore used a 20 Myr window, selected empirically to capture localized dynamics rather than whole-record trends. Indicator values are plotted at the end of each window; the horizontal offset between the start of the input record and the onset of the indicator curve corresponds to this 20 Myr aggregation.

Detrending and sensitivity. Lag-1 autocorrelation is sensitive to non-stationarity, so each series was detrended prior to EWS estimation. Detrending settings were selected via numerical experiments to remove visible residual trends while preserving transition-scale variability. We then performed empirical sensitivity tests by varying both the sliding-window length and detrending parameters; the resulting spread across runs provides the uncertainty ranges shown alongside the average EWS curves.

S1.2 Alignment of Alroy (3T) and Foote (PC) vulnerability series.

To compare vulnerability reconstructions derived from Alroy's three-timer (3T) framework [1] and Foote's per-capita (PC) framework [2] (Fig. S1), the 3T-based vulnerability series was interpolated to the PC age grid and a near-constant vertical offset was estimated. In the convention used here, the interpolated 3T series is lower than the PC series by $\Delta \approx 0.428$ (mean PC minus 3T), so for direct visual comparison of excursions we also show an offset-corrected version obtained by shifting the 3T series upward by $+\Delta$. Agreement statistics and alignment variants are summarized in Table S1. In Table S1c, the "alternative alignment variant" refers to the same procedure applied over the overlap interval with complete data coverage (hence the reduced sample size).

For joint display with vulnerability, the $p\mathrm{CO}_2$ curve in Fig. S1a is rescaled as $(p\mathrm{CO}_2/\langle p\mathrm{CO}_2\rangle) - 1$ (blue trace), as indicated in the figure caption. This transformation is for visualization only and is not used in statistical calculations.

Supplementary Table S1: **Vulnerability alignment statistics.** Pearson correlations between Foote PC and Alroy 3T vulnerability after interpolation to the PC age grid and (where indicated) offset correction. A constant vertical offset does not alter the Pearson correlation; rows (a) and (b) are therefore expected to be identical.

Comparison	Pearson r	Notes
(a) Foote PC vs. interpolated Alroy 3T	+0.78	$p < 0.01$, $N = 74$
(b) Foote PC vs. interpolated + shifted Alroy 3T ($+\Delta$, $\Delta = 0.428$)	+0.78	$p < 0.01$, $N = 74$
(c) Foote PC vs. alternative alignment variant	+0.84	$p < 0.01$, $N = 61$

S2 Recurrence diagnostics and regime partitioning

S2.1 Recurrence diagnostics.

Recurrence analysis is well suited to nonstationary, noisy, and intermittently switching systems and has been used to detect regime shifts and time-dependent synchronization between components in uni- and multivariate paleo-records [3–6].

$$R_{ij}(\varepsilon) \;=\; \Theta\big(\varepsilon - \|x_i - x_j\|\big), \qquad i, j = 1, \ldots, N \tag{S1}$$

$$\mathrm{JR}_{ij} \;=\; R^{(x)}_{ij}(\varepsilon_x) \,\cdot\, R^{(y)}_{ij}(\varepsilon_y) \tag{S2}$$

In Eq. (S1), $R(\varepsilon)$ is the *square* $N \times N$ *binary* (auto)recurrence matrix with entries $R_{ij} \in \{0, 1\}$; Θ is the Heaviside step function, which yields 1 when the inter-state distance is $\leq \varepsilon$ (chosen dissimilarity threshold, which in the present application is autotuned so that the recurrence plot attains a prescribed percentage of recurrences) and 0 otherwise; x_i is the system state at time index i; and $\|\cdot\|$ denotes the chosen norm. For this study we build recurrence plots (RPs) without embedding on the smoothed isotope series, so x_i is scalar and the distance reduces to $|x_i - x_j|$. In Eq. (S2), $\varepsilon_x, \varepsilon_y$ are thresholds for the two univariate RPs and the Hadamard product keeps only simultaneous (coincident) recurrences.

Complementary to the joint recurrence plot (JRP), a bivariate autorecurrence is computed directly on the normalized pair (δ^{13}C, δ^{18}O), producing a single 2D RP that highlights jointly occupied regions of isotope

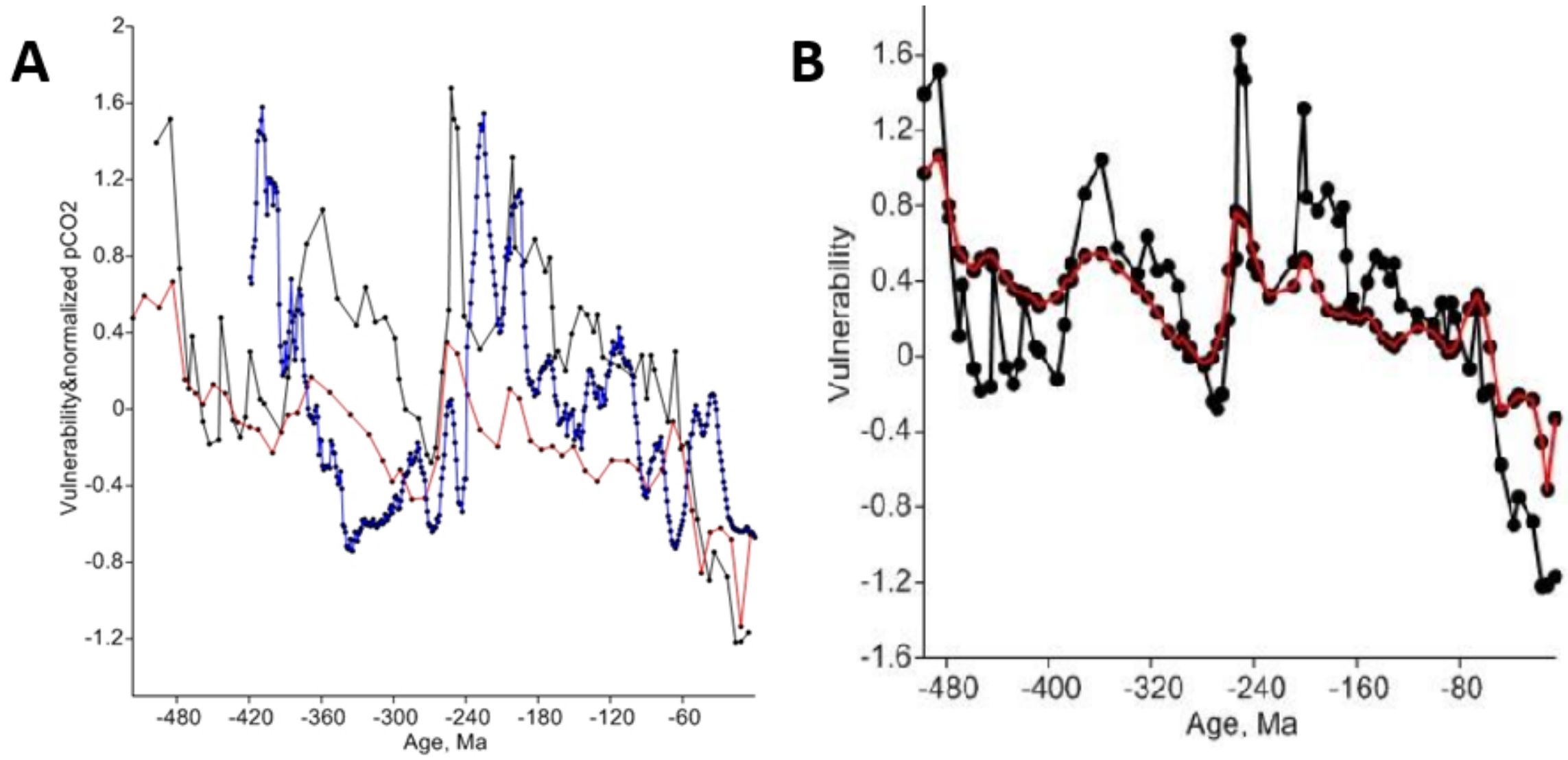

Supplementary Fig. S1: **Comparison of vulnerability time series.** **(a)** Vulnerability time series: Alroy 3T (red) and Foote PC (black), with rescaled pCO_2 shown for visual comparison (blue; $(pCO_2/\langle pCO_2\rangle) - 1$). **(b)** Alroy 3T vulnerability interpolated to the Foote PC age grid (red) and vertically shifted by $+\Delta$ (with $\Delta = 0.428$) to align baselines for comparison of excursions; the Foote PC series on the same grid is shown for reference (black).

state space. Here "normalized" means that each isotope series is standardized to zero mean and unit variance over the analyzed interval before forming the 2D state $z_i = (z_i^{(C)}, z_i^{(O)})$; distances are then computed with the Euclidean norm $\|z_i - z_j\|_2$.

For the constructions above we use:

- **Univariate RPs** for δ^{13}C and δ^{18}O constructed from the pre-processed series (no embedding), with thresholds chosen to enforce a recurrence rate, RR = 20%.
- **JRP** formed as the element-wise (Hadamard) product of the two univariate RPs, retaining only coincident recurrences, with thresholds chosen to enforce RR = 10%.
- **Bivariate autorecurrence** on the normalized (δ^{13}C, δ^{18}O) pair, with thresholds chosen to enforce RR = 10%.

All RP/JRP calculations were implemented using custom `Perl` scripts included in the deposited code package cited in the Code Availability section.

Block-like textures along the line of identity indicate quasi-stationary intervals where isotope states recur over multi-Myr spans, whereas abrupt changes in texture or extended low-recurrence ("white") regions mark transitions. Because JRPs retain only coincident recurrences, boundaries appear sharper when both proxies shift together. We use these coordinated changes to define a seven-state partition at the scale range considered; for clarity in the main text we consolidate this finer partition into the five mega-climate states reported there.

S2.2 Seven-state partition and consolidation

The seven-state partition (used for finer-scale reporting) is:

1. **Cambrian–Upper Ordovician** (538.5–447.3 Ma).
2. **Upper Ordovician–lowermost Carboniferous** (447.3–357.9 Ma).
3. **Carboniferous–Middle Triassic** (357.9–238.8 Ma).
4. **late Middle–Upper Triassic** (238.8–211.4 Ma).
5. **Upper Triassic–Paleocene** (211.4–56.8 Ma).
6. **latest Paleocene–Eocene** (56.8–38.3 Ma).
7. **late Eocene–Quaternary** (38.3–0 Ma).

In the main text, states (4) and (5) are combined into a single **Mesozoic–Lower Paleogene** mega-state, and states (6) and (7) are combined into a single **Cenozoic** mega-state (states (1)–(3) map directly to the first three mega-states).

The univariate autorecurrence plots (Fig. S2), the bivariate autorecurrence (Fig. S3a), and the joint recurrence plot (Fig. S3b) for δ^{18}O and δ^{13}C show major structural shifts in proxy dynamics and their coordinated transitions. Because climate variability is self-similar across scales [7], transitions are expected at multiple characteristic time scales. Here we focus on changes on the order of tens to ~100 Myr, motivated by the fact that biodiversity dynamics shows its strongest contrasts in diversity states on comparable time scales [8]. At this scale range, coordinated changes in the JRP (Fig. S3b), supported by the bivariate autorecurrence (Fig. S3a), provide the working boundaries for the seven-state partition above.

The δ^{13}C autorecurrence plot (Fig. S2a) shows two first-order change-points: (i) at ~447 Ma, separating the Cambrian–Upper Ordovician interval from the remainder of the Paleozoic and the Mesozoic up to the Eocene; and (ii) at ~57 Ma, marking the transition to the post-Paleocene Cenozoic. Within the repetitive background from the Upper Ordovician to the Paleocene, the Cambrian–Upper Ordovician and the post-Paleocene Cenozoic appear as distinct end-member states. The ”chessboard” texture within the quasi-stable Upper Ordovician–Paleocene interval indicates reversible transitions between ~100 Myr-scale states on the background of relative stasis in long-term mean δ^{13}C.

The δ^{18}O autorecurrence plot (Fig. S2b) exhibits an intermittently trending character with a clearer sequence of state transitions. A transition near ~447 Ma aligns with the shift seen in δ^{13}C. A further transition in the Triassic (~230–240 Ma) separates the Paleozoic–Lower Triassic state from the Mesozoic-to-Eocene state, followed by the transition to the post-Paleocene Cenozoic near ~57 Ma.

The bivariate autorecurrence plot of the normalized (δ^{13}C, δ^{18}O) pair (Fig. S3a) yields patterns closely matching those in the JRP constructed from separate univariate autorecurrence plots (Fig. S3b). Across the chosen scale range, the block structure supports the coordinated seven-state δ^{13}C–δ^{18}O partition through the Phanerozoic.

Finally, the triple joint recurrence plot of vulnerability with δ^{13}C and δ^{18}O (Fig. S4) shows a coordination pattern similar to that of the δ^{13}C–δ^{18}O recurrence diagnostics, consistent with coupled evolution of all three variables. The strongest contrasts again separate the lower Paleozoic (Cambrian through Upper Ordovician) and the post-Paleocene Cenozoic. The most prominent transition (a cross-like structure near the center of the triple JRP) coincides with the Permian–Triassic interval, indicating that long timescale climate–carbon cycle transitions are reflected in macroevolutionary stress patterns.

S2.3 Vulnerability stratified by Haggis bins (mega-climate)

The dynamics of macroevolutionary vulnerability can be further explored by subdividing the record into the Haggis bins (mega-climate regimes) determined from the coordinated δ^{13}C–δ^{18}O recurrence structure, thus highlighting potential connections of long-term temperature and carbon-cycle states (Fig. S5). The correlation between the temperature proxy ($-\delta^{18}$O; higher values indicate warmer conditions) and vulnerability within these time slices is inconsistent, in contrast to a significant positive association at the scale of the full Phanerozoic for both the Foote per-capita (PC) reconstruction [2] and the Alroy three-timer (3T) reconstruction [1] (r = 0.38–0.42, $p < 0.01$). This scale dependence supports the interpretation that elevated background biotic stress (high turnover combined with depressed diversity) is primarily associated with long-time-scale warm mega-climate background conditions (10s–100s of Myr), whereas epoch- and stage-scale conditions can be associated with a wide range of vulnerability levels.

Two long intervals stand out in Fig. S5: the Cambrian greenhouse shows consistently high vulnerability (comparable to the Permian–Triassic and Early Triassic peaks), whereas the overall cool Cenozoic has the lowest vulnerability. This contrast is consistent with positive scaling of mega-climate variability [7, 8], which implies that the largest background temperature differences emerge on the longest time scales. These patterns may also help contextualize the extremely high macroevolutionary volatility in the early Paleozoic and low volatility in the Cenozoic [9], where volatility is defined as $(p + q)/D$ (closely related to vulnerability but without mean-diversity standardization and on an arithmetic rather than logarithmic scale). While long-term biological sorting may contribute to declining volatility, the coincidence of volatile and hot climatic regimes with high vulnerability (and conversely cold states with low vulnerability) points to the importance of physical forcing in setting the background biotic stress regime.

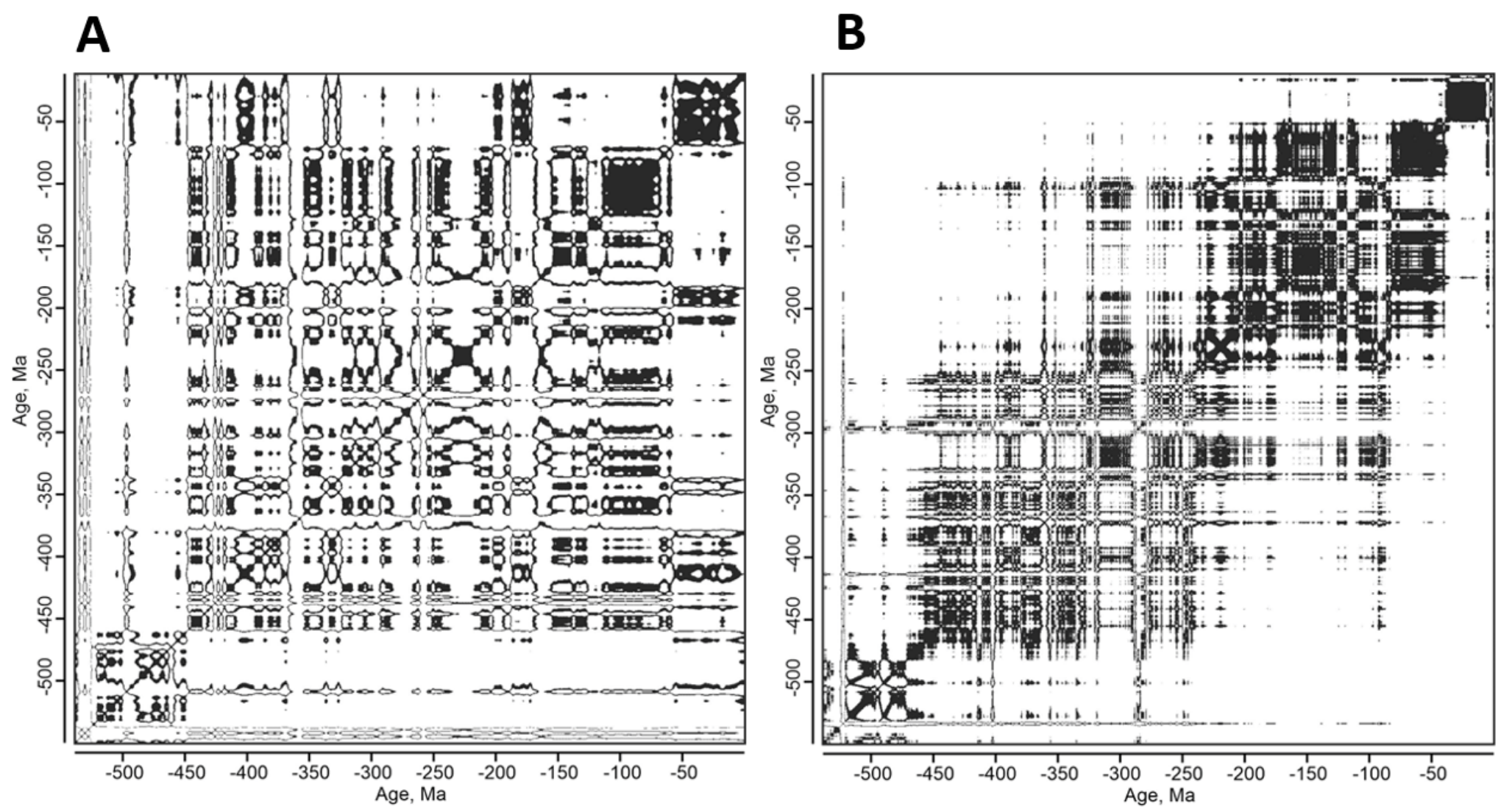

Supplementary Fig. S2: **Autorecurrence structure of δ^{13}C and δ^{18}O.** Autorecurrence plots of **(a)** δ^{13}C and **(b)** δ^{18}O at fixed recurrence rate (RR = 20%). Block-like textures indicate quasi-stationary intervals; low-recurrence bands indicate transitions.

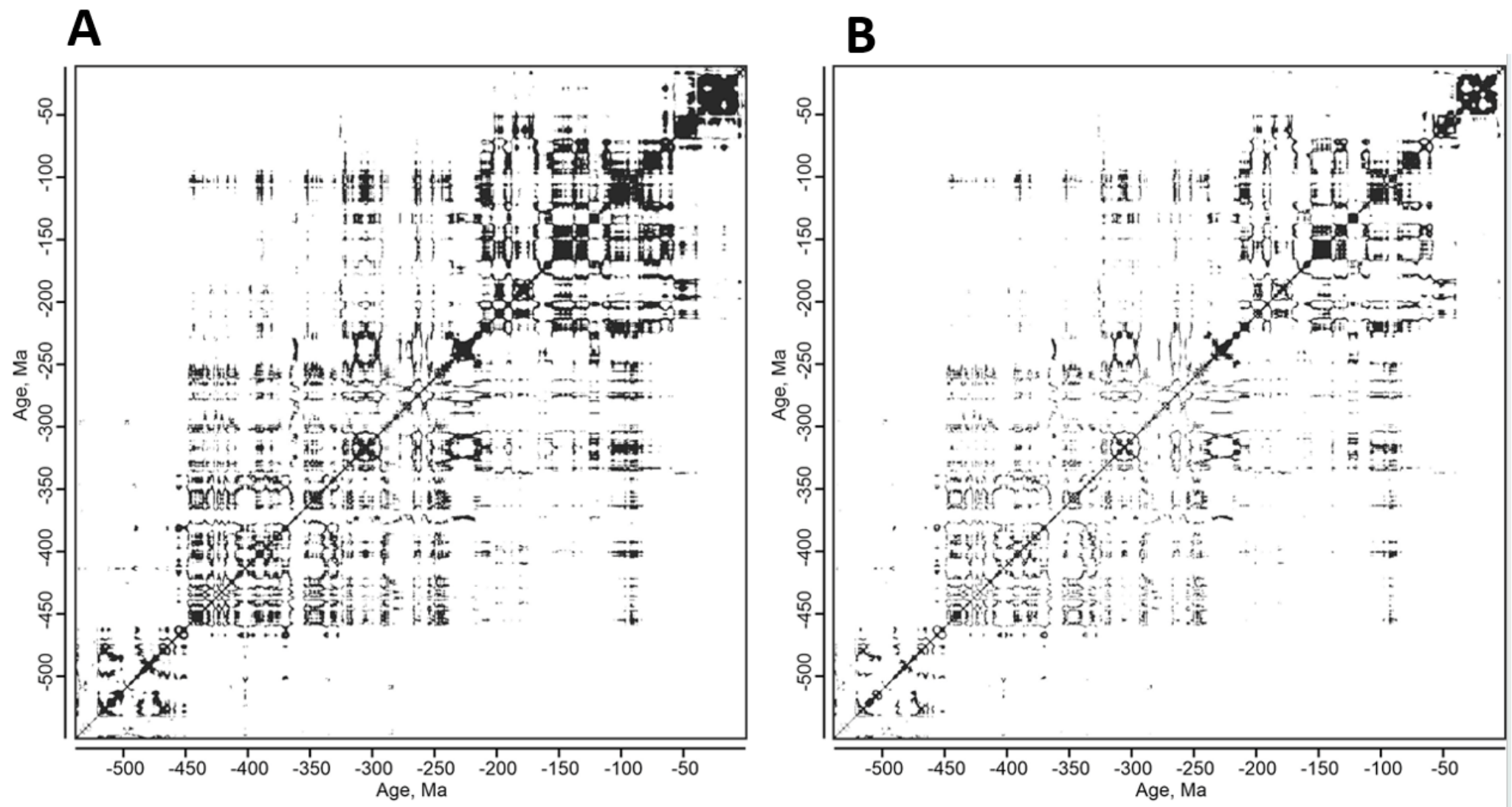

Supplementary Fig. S3: **Bivariate autorecurrence and joint recurrence of δ^{13}C and δ^{18}O.** **(a)** Bivariate autorecurrence of normalized (δ^{13}C, δ^{18}O) (RR = 10%). **(b)** JRP constructed from the corresponding univariate RPs, retaining coincident recurrences after thresholding as described in Section S2.1.

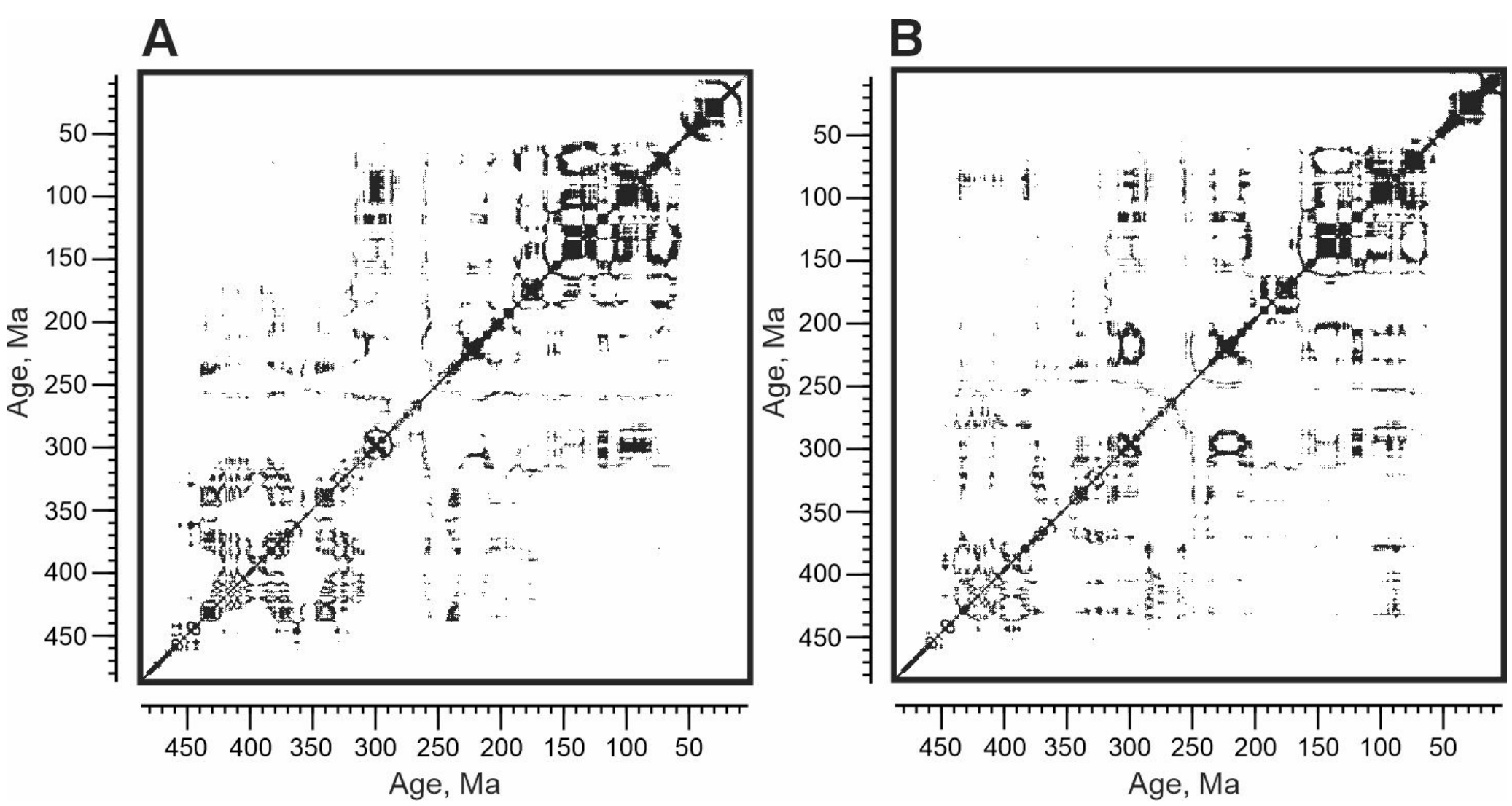

Supplementary Fig. S4: **Triple joint recurrence of vulnerability and stable-isotope proxies.** Triple joint recurrence plots linking vulnerability with δ^{13}C and δ^{18}O, highlighting intervals of simultaneous recurrence across all three variables. **(a)** Vulnerability computed from Alroy's 3T turnover/diversity reconstruction. **(b)** Vulnerability computed from Foote's PC turnover/diversity reconstruction.

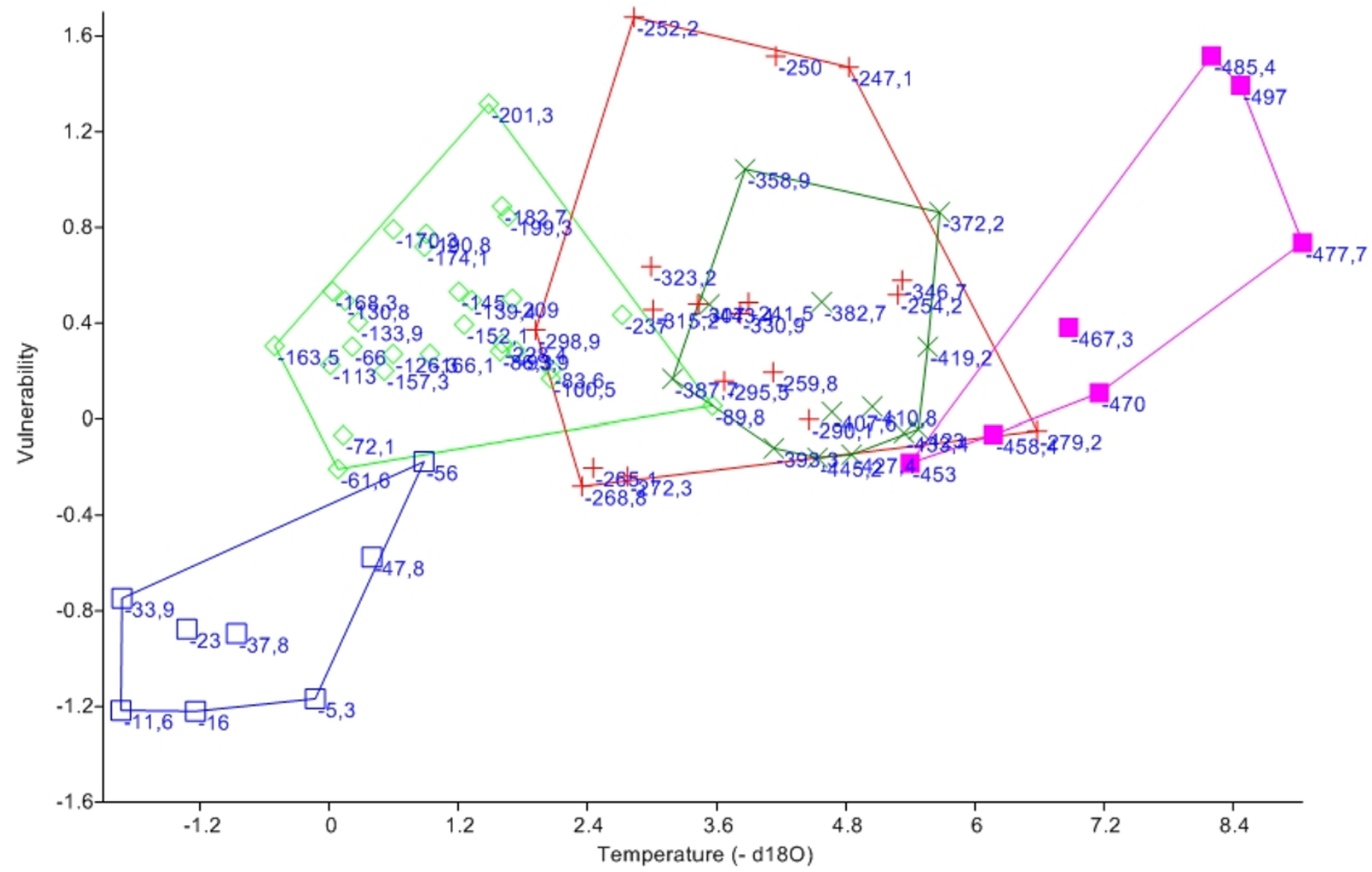

Supplementary Fig. S5: **Vulnerability across Haggis time bins versus temperature proxy.** Convex hulls summarize vulnerability within each Haggis time slice (bin), plotted against $-\delta^{18}$O (x-axis; higher values indicate warmer conditions).

S3 The Conceptual Model

S3.1 Governing equations and equilibrium structure

Our conceptual model follows the climate–carbon system developed in [10], in which global mean atmospheric CO_2 concentration C (treated as pCO_2 in the proxy reconstructions) and surface air temperature T evolve according to coupled ordinary differential equations for the carbon cycle and the planetary energy balance. The carbon equation is

$$\frac{\mathrm{d}C}{\mathrm{d}t} \;=\; F_1(C,T;\mathbf{P}) \;=\; V - W(T)\Big(\frac{C}{C_0}\Big)^{\beta}, \tag{S3}$$

where V is a long-term volcanic–biospheric source term, C_0 is a reference CO_2 level, $\beta \approx 1/2$, and $W(T)$ is a temperature-dependent silicate weathering function. When impulsive forcing is considered (Section S3.4), it is represented as additional pulse components in the source term. Here, we use

$$W(T) \;=\; \Big[\tfrac{1}{2} + \frac{1}{\pi}\arctan\Big(\frac{T-273}{5}\Big)\Big]\exp\big[-k(T-288)\big], \tag{S4}$$

so that weathering is negligible in very cold climates, increases rapidly across the freezing point, and decays again at very high temperatures. This choice reproduces the asymmetric, excitable behavior of the carbon cycle described in [10] and underlies the existence of distinct cool and warm climate branches.

The temperature equation is an energy-balance model of Budyko–Sellers type with temperature-dependent

albedo and logarithmic greenhouse forcing, written as

$$\frac{\mathrm{d}T}{\mathrm{d}t} \;=\; F_2(C,T) \;=\; 1 - A(T;T_{\mathrm{alb}}) + \varepsilon\Big(\frac{T}{T_0}\Big)^4 + b\,\log\Big(\frac{C}{C_0}\Big), \tag{S5}$$

where $A(T;T_{\mathrm{alb}})$ is a smooth sigmoidal ice–albedo function with characteristic transition temperature T_{alb}, T_0 is a reference temperature, ε sets effective long-wave emission, and b controls the SAT response to $\log(C/C_0)$. Unless stated otherwise, any additional shape parameters defining $A(T;T_{\mathrm{alb}})$ are treated as fixed.

On the multi-Myr time scales of interest, energy-balance adjustment is fast compared with the carbon cycle and tectonic forcing. In the main analysis we therefore assume that the climate is close to thermal equilibrium and solve the steady form $F_2(C,T) = 0$ for T at each time step, with C given by the proxy-based $p\mathrm{CO_2}$ history:

$$0 \;=\; 1 - A(T;T_{\mathrm{alb}}) + \varepsilon\Big(\frac{T}{T_0}\Big)^4 + b\,\log\Big(\frac{C}{C_0}\Big). \tag{S6}$$

Because the equilibrium condition can admit multiple solutions T for fixed C, we select one of the possible equilibrium branches $T_i(C)$ as described below.

S3.2 Discrete-time formulation and parameter estimation

For parameter estimation, we use a discrete-time approximation to the carbon equation on a uniform grid $t_j = t_0 + j\Delta t$ with $\Delta t = 1$ Myr:

$$C(t_{j+1}) \;=\; C(t_j) + R_C\,F_1\big(C(t_j),T(t_j);\mathbf{P}\big) + \varepsilon_C(t_j), \tag{S7}$$

where R_C rescales the continuous-time rate and $\varepsilon_C(t_j)$ is a Gaussian noise term representing unresolved variability. In this calibration step, $C(t_j)$ and $T(t_j)$ are taken from the $p\mathrm{CO_2}$ and SAT reconstructions on the target interval.

Defining the one-step residual as

$$Q\big(X(t_j),\mathbf{P}\big) \;=\; C(t_{j+1}) - C(t_j) - R_C\,F_1\big(C(t_j),T(t_j);\mathbf{P}\big), \tag{S8}$$

and assuming independent Gaussian errors, the likelihood of the trajectory $X(t_j) = (C(t_j), C(t_{j+1}), T(t_j))^\top$ is proportional to

$$\mathcal{L}(\mathbf{P}) \;\propto\; \prod_j \exp\left(-\frac{Q\big(X(t_j),\mathbf{P}\big)^2}{2\sigma^2}\right), \tag{S9}$$

which (for fixed σ) we maximize to obtain maximum-likelihood estimates of $\mathbf{P}$ on a given time interval. Equivalently, this corresponds to least-squares minimization of $\sum_j Q(X(t_j),\mathbf{P})^2$.

Parameter vector. We denote by $\mathbf{P}$ the set of parameters estimated in the discrete-time formulation. In our analysis we fit, within each Haggis time window, the energy-balance parameters $(b, C_0, T_{\mathrm{alb}})$ together with selected carbon-cycle parameters (e.g. V and k), while treating the remaining parameters in Eqs. (S3)–(S5) (including β, T_0, ε, and any additional shape parameters defining A) as fixed. If the discrete-time rate factor R_C is estimated, it is included in $\mathbf{P}$. We apply this framework separately to the four Haggis bins for which both SAT and $p\mathrm{CO_2}$ reconstructions are available.

S3.3 Reconstruction of SAT–pCO_2 states

Once parameters are calibrated in each time bin window, we reconstruct 1-Myr trajectories of SAT and pCO_2 by evaluating the equilibrium condition $F_2(C(t_j), T(t_j)) = 0$ at each time step, with $C(t_j)$ taken from the proxy pCO_2 series. When multiple equilibrium branches exist, we select the branch that lies closest to the empirical SAT value at that time. The collection of points $\big(C(t_j), T(t_j)\big)$ forms a reconstructed trajectory in SAT–pCO_2 space that can be compared directly with the empirical SAT–pCO_2 structure in the main paper.

S3.4 Representation of episodic perturbations in the carbon source term

Discrete geological events can be represented as transient perturbations of the carbon source term. In the continuous formulation we write the source as a background term plus a sum of Gaussian pulses and substitute $V \to V(t)$ in Eq. (S3):

$$V(t) \;=\; V_0 + \sum_k A_k \exp\left[-\frac{(t-\tau_k)^2}{2\lambda_k^2}\right], \qquad \text{(S10)}$$

where A_k is the amplitude, τ_k the central time, and λ_k the characteristic duration of the k-th event. Positive pulses represent transient CO_2 injections (e.g., LIP-scale volcanism), whereas negative pulses can be used as a phenomenological representation of rapid carbon drawdown and associated cooling; such negative pulses may be consistent with large impact events, but we treat them here as a generic class of short-lived perturbations.

In a broader modeling workflow, candidate pulse components can be tested using the discrete-time formulation in Eq. (S7) together with model selection based on the Akaike information criterion (AIC). In brief, the source term is represented as a smooth background plus a small number of candidate pulses, parameters are fitted under the same discrete-time Gaussian likelihood, and AIC is used to balance improved fit against added complexity. This yields estimates of pulse timing, amplitude, and duration and provides a quantitative test of whether a localized injection-like or draw-down-like component is supported relative to a no-pulse model.

In the analysis presented here we do not re-estimate pulse parameters from scratch. Instead, we use the Gaussian-pulse form in Eq. (S10) with parameter ranges chosen to produce Myr-scale excursions comparable to those observed in the proxy records, and we place pulses at established candidate event times (e.g., the Siberian Traps at $\sim$ 252 Ma and the Central Atlantic Magmatic Province at $\sim$ 201 Ma). For a short-lived negative perturbation we use a pulse centered at 66 Ma; we treat this as a convenient representation of a rapid draw-down/cooling event, noting that it coincides with the Cretaceous–Paleogene boundary and may be consistent with an impact-related perturbation. However, we do not attribute causality to any specific mechanism in this representation.

S3.5 Construction of the discrete attractor record

To summarize the continuous SAT–pCO_2 reconstruction in a discrete form without imposing a temperature-ranking scheme, we construct an attractor record $A(t) \in \{\mathrm{A}_1, \ldots, \mathrm{A}_5\}$. Here A_k denotes the k-th equilibrium solution (attractor branch) of the equilibrium constraint in Eq. (S6) available at a given time within each time window.

Starting from the proxy-driven equilibrium reconstruction, at each time step t_j we evaluate the equilibrium constraint and identify all coexisting equilibrium solutions by scanning in T and detecting zero crossings of the equilibrium residual. This yields a set of candidate equilibria $\{T^{(k)}(t_j), C^{(k)}(t_j)\}_{k=1}^{K(t_j)}$, with $K(t_j) \le 5$.

We then select a single equilibrium solution by choosing the candidate $T^{(k)}(t_j)$ that lies closest to the reconstructed paleotemperature at that time. The discrete record is defined as

$$A(t_j) = \mathrm{A}_k \quad \text{if} \quad (T^{(k)}(t_j), C^{(k)}(t_j)) \text{ is the selected equilibrium solution at } t_j. \tag{S11}$$

This produces the stepwise series shown in Fig. 4 of the main text. Extended plateaus correspond to sustained residence on one equilibrium branch, whereas sharp changes indicate switching between coexisting equilibria and/or reorganization of the equilibrium structure under transient forcing. In this representation, discrete changes are interpreted as transitions between equilibrium solutions of the coupled system, rather than as movement between pre-defined temperature bands.

S4 Limitations and consistency of the approach

Several limitations remain and motivate conservative interpretation of regime boundaries and biological associations:

- **Time-scale separation (EWS vs. trend–fluctuation frameworks).** Most early warning signals (EWS) rely on an assumed separation between *fast* stochastic fluctuations and *slow* drift in boundary conditions. In deep time this separation is harder to justify operationally because sampling is irregular, proxy uncertainty is time-dependent, and chronological error can redistribute variance across frequencies. For this reason we do not interpret EWS as a literal forecast with a fixed lead time; instead, we treat them as diagnostics of *system-level* changes in fluctuation structure that accompany regime reorganization. This stance also clarifies the relation of sequential approaches to extinction risk that decompose trajectories into "trend" and "fluctuations" (e.g., [11]): such decompositions can be informative but often leave the time-scale of the trend and the bandwidth of the fluctuations implicit. Here, EWSs are used primarily to detect whether the *background* state is changing, and we place most weight on signals that are consistent *across proxies* and robust to reasonable pre-processing choices.

- **Proxy heterogeneity and chronology.** Deep-time records are nonstationary and heterogeneous across archives, and age uncertainty can blur the timing of rapid transitions or compress multi-pulse perturbation intervals. We therefore interpret bin boundaries at multi-Myr resolution and treat them as approximate within chronological uncertainties. This has two practical implications: (i) boundaries are best viewed as *transition zones* rather than instantaneous change-points, and (ii) apparent lags between abiotic transitions and biotic responses should not be over-interpreted without explicit age-uncertainty propagation. Where possible, we lean on *coordinated* changes (joint behavior in δ^{13}C and δ^{18}O) because simultaneous structure across proxies is less likely to arise from sampling artefacts than a signal in a single series.

- **Composite vs. local records.** The analytical framework presented here is designed for interregional or global composite records, and should not be transferred directly to individual local stratigraphic sections without additional checks. Local sections are intrinsically incomplete, and section-specific hiatuses, unconformities, or facies shifts can generate an artificial concentration of apparent changes across multiple variables, potentially mimicking synchronous regime shifts. By synthesizing multiple widely distributed records, global compilations help mitigate the influence of these local stratigraphic gaps on inferred transition structure. Consequently, any application of recurrence- or early-warning-style analyses at the scale of a single local section would require explicit treatment of section completeness, sediment accumulation, and correlation structure before transition timings could be interpreted with confidence.

- **Biological-response heterogeneity (boundaries vs. impulsive events).** The correspondence between mega-climate boundaries and mass extinctions is not one-to-one. A natural interpretation is that there are (at least) two end-member classes of extreme events: (i) *Earth system transition* events in which biotic loss is amplified because the system is already reorganizing—feedbacks are shifting and buffering changes, so the same external stressor can push the coupled climate–carbon system across an internal threshold; and (ii) *impulsive* events dominated by a short, high-magnitude perturbation, whose impact depends less on the background state's proximity to a boundary. In this framing, coincidence with a boundary is not a requirement for high extinction intensity, but it may help explain why some crises show broader, more prolonged environmental disruption whereas others appear sharper or more event-like. This perspective motivates treating mega-climate states as *conditioning contexts* that modulate the severity, selectivity, and recovery dynamics of biotic responses, rather than as deterministic triggers for every extinction pulse.

- **Long-term vulnerability trends and the role of modeling.** Vulnerability exhibits a systematic long-term change across the Phanerozoic, which could reflect (a) shifts in the distribution of background climate states and their typical variability, (b) secular evolution in ecosystem structure and self-stabilizing properties (e.g., changes in ecological buffering, spatial connectivity, or the standing diversity baseline against which turnover acts), or (c) a combination of both. Disentangling these effects is where modeling is useful: a reduced climate–carbon model can generate controlled counterfactuals in which short-time stressors (e.g., pulse-like perturbations) are applied to different background equilibria, allowing explicit tests of whether identical perturbations produce systematically different extinction-sized responses depending on the mega-climate state. Conversely, mismatches between model expectations (state-dependent sensitivity) and realized fossil responses can help identify events dominated by non-climatic mechanisms, compound stressors, or strong biological contingencies. In this sense, the model is not used to predict individual extinctions, but to separate *background-state conditioning* from *stressor magnitude and timing*, and to clarify which aspects of the extinction record are consistent with regime-dependent Earth system sensitivity.

S5 Punctuated early-warning structure in graptoloid diversity across the Ordovician–Silurian transition

The global standing graptoloid species diversity series in Fig. S6a is the published CONOP (constrained optimization of stratigraphic ranges) reconstruction reported in [12]. Here we use a digitized version of that curve as input for EWS calculations. The record shows a pronounced boom–bust pattern through the Ordovician and Silurian, including a severe Late Ordovician decline associated with the Late Ordovician mass extinction (LOME) [12]. Across the middle-to-upper Ordovician transition, this series marks a step change in extinction regime in marine plankton [12], and subsequent turnover shows longer-period pacing consistent with Milankovitch "grand cycles" [13].

To assess whether this high-resolution biodiversity record contains dynamical precursors consistent with a regime shift, we computed a sliding-window lag-1 autocorrelation indicator (ACF_1) along the diversity time series (Fig. S6b). The ACF_1 trajectory exhibits two dominant decrease–increase excursions during the Late Ordovician: a first excursion near ~455 Ma (early Katian) and a second near ~445 Ma (Hirnantian) (Fig. S6b). Rather than a single monotonic increase in persistence, this repeated decrease–recovery structure is consistent with a punctuated, multi-episode transition in which the system transiently enters a low-persistence regime before reorganizing toward a new diversity state. This interpretation is compatible with the view that the

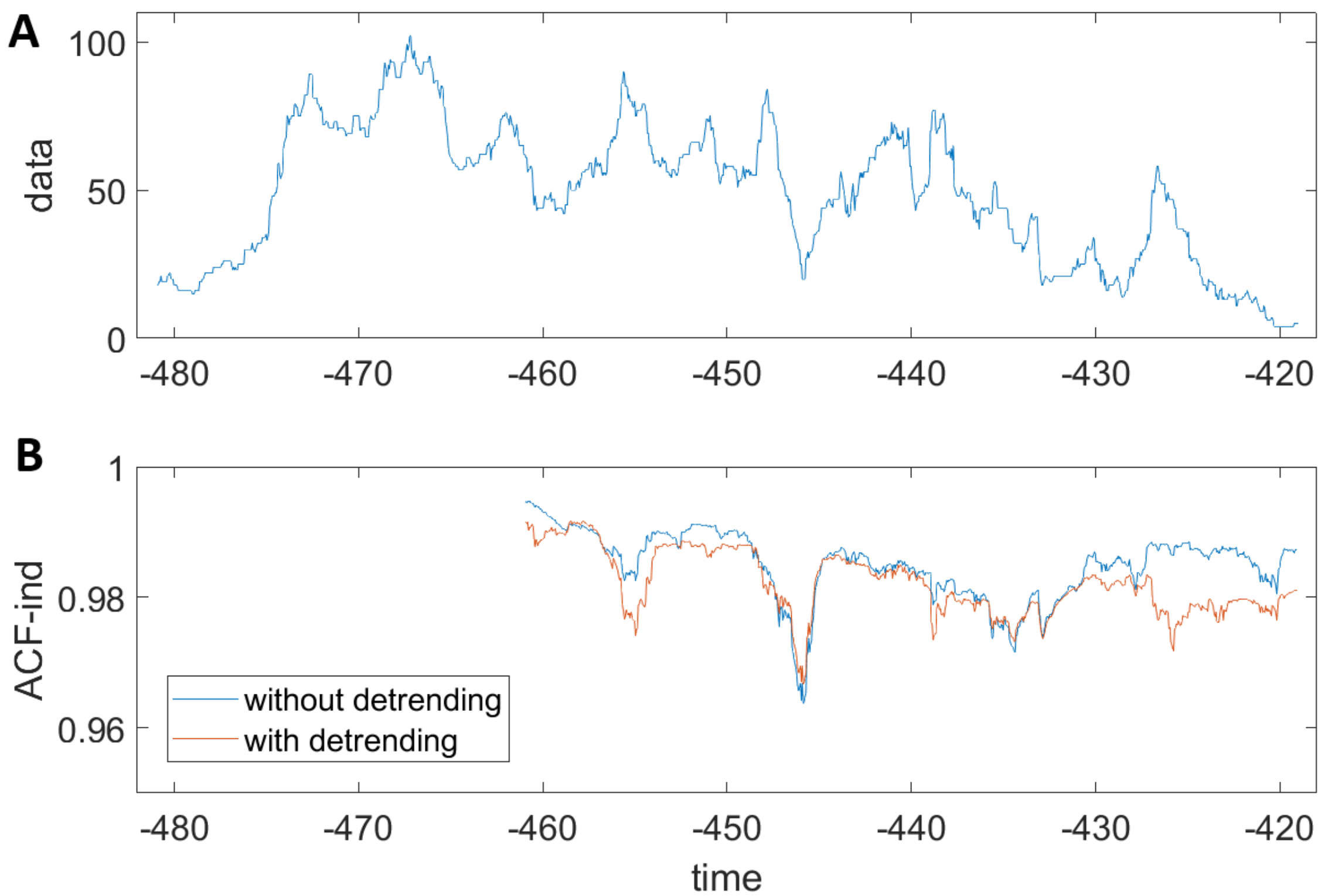

Supplementary Fig. S6: **Graptoloid diversity and lag-1 autocorrelation across the Ordovician–Silurian transition. (a)** Global standing graptoloid species diversity reconstructed using CONOP. **(b)** Windowed lag-1 autocorrelation (ACF_1; AR(1) coefficient) computed along the diversity record, shown for the raw series and after detrending. Two prominent excursions occur during the Late Ordovician (early Katian and Hirnantian), consistent with a punctuated, multi-phase disruption rather than a single instantaneous transition.

LOME was prolonged and punctuated, potentially involving multiple extinction pulses nested within an extended interval of several Myr [14].

The graptoloid record therefore provides an independent, high-resolution illustration that major Ordovician–Silurian biodiversity reorganizations can unfold as a cascade of discrete dynamical episodes rather than a single instantaneous change-point, supporting the interpretation that boundaries near ∼447 Ma are best treated as multi-Myr transition zones [12, 14].

S6 Cross-diagnostic transition timing

Supplementary Table S2 *(attached as a separate file)* provides the chronological crosswalk used for timing comparisons across independent diagnostics. At 10 Myr spacing through the Phanerozoic, it lists stratigraphic context together with (i) published qualitative climate-state assignments (as indicated by the column headings), (ii) Haggis-bin membership inferred from both the SAT–pCO_2 pinch-point analysis and the $\delta^{18}O$–$\delta^{13}C$ joint-recurrence analysis (and the reconciled final bin), and (iii) the corresponding model attractor/branch

label. The table also records change-point/early-warning detections and major external markers (CIEs, robustly dated LIP intervals, mass-extinction intervals, and vulnerability summaries), enabling direct visual alignment of inferred transitions across proxies, model reconstructions, and event compilations.

References

1. Alroy, J. Accurate and precise estimates of origination and extinction rates. *Paleobiology* **40,** 374–397 (2014).
2. Foote, M. Diversity-Dependent Diversification in the History of Marine Animals. *The American Naturalist* **201,** 680–693 (2023).
3. Westerhold, T. *et al.* An astronomically dated record of Earth's climate and its predictability over the last 66 million years. *Science* **369,** 1383–1387 (2020).
4. Marwan, N., Romano, M. C., Thiel, M. & Kurths, J. Recurrence plots for the analysis of complex systems. *Physics Reports* **438 (5–6),** 237–329 (2007).
5. Spiridonov, A. *et al.* Ultra-high resolution multivariate record and multiscale causal analysis of Pridoli (late Silurian): implications for global stratigraphy, turnover events, and climate-biota interactions. *Gondwana Research* **86,** 222–249 (2020).
6. Stankevič, R., Venckutė-Aleksienė, A., Radzevičius, S. & Spiridonov, A. Phytoplankton and zooplankton paleocommunity change before and during the onset of the Lau Extinction Event (Ludlow, Silurian). *Marine Micropaleontology* **189,** 102368 (2024).
7. Lovejoy, S. A voyage through scales, a missing quadrillion and why the climate is not what you expect. *Climate Dynamics* **44,** 3187–3210 (2015).
8. Spiridonov, A. & Lovejoy, S. Life rather than climate influences diversity at scales greater than 40 million years. *Nature* **607,** 307–312 (July 2022).
9. Lieberman, B. & Melott, A. Declining volatility, a general property of disparate systems: from fossils, to stocks, to the stars. *Palaeontology* **56(6),** 1297–1304 (2013).
10. Arnscheidt, C. & Rothman, D. Routes to global glaciation. *Proc. R. Soc. A* **476,** 20200303 (2020).
11. Flügel, E. & Kiessling, W. *Patterns of Phanerozoic reef crises* in *Phanerozoic Reef Patterns* (eds Kiessling, W., Flügel, E. & Golonka, J.) (2002).
12. Crampton, J. S., Cooper, R. A., Sadler, P. M. & Foote, M. Greenhouse–icehouse transition in the Late Ordovician marks a step change in extinction regime in the marine plankton. *Proceedings of the National Academy of Sciences* **113,** 1498–1503 (2016).
13. Crampton, J. S. *et al.* Pacing of Paleozoic macroevolutionary rates by Milankovitch grand cycles. *Proceedings of the National Academy of Sciences* **115,** 5686–5691 (2018).
14. Rasmussen, C. M., Vandenbroucke, T. R. A., Nogues-Bravo, D. & Finnegan, S. Was the Late Ordovician mass extinction truly exceptional? *Trends in Ecology & Evolution* **38,** 812–821 (2023).

TIME (Ma)	Geol Era	Geol Period or Epoch	Judd et al 2024 [10]	Vérard 2024 [22]	Marcilly et al 2022 [13]	Scotese et al 2021 [18]	McKenzie et al 2016 [14]	Link 2009 [12]	d18O–d13C joint recurrence plot mega-climate states	Mega-climate states summarized into Haggis bins	Conceptual climate-carbon model attractors	EWS d18O	EWS d13C	CIEs [5]	LIPs ("robust" dates only from [9])	Continental Arc Volcanism events (CAVs; broadly from [14])	ME intervals [2][3][8]	Vulnerability (raw)	Major Transitions in Phanerozoic Biosphere and Climate
0		Quat-Neog															Plio		
10		Neogene	cool-cold	icehouse	cold	cold	icehouse	icehouse	7		A3 (0 Ma)			+	Columbia River Basalts (16.74-14.78 Ma main phase; may last until 5-6 Ma) Ethiopia-Yemen (34.43-15.6 Ma)				~ evolution and demise of mammalian megafauna (~ 40-0.025 Ma) [20]
20	Cenozoic		transitional							5		~10-30 Ma		+		few CAVs - CENO		neg	
30			transitional-cool										~ 30-50 Ma	+	Ethiopia-Yemen (34.43-15.6 Ma)		PETM		
40			transitional-warm						6		A4 (48 Ma); A5 (49 Ma)			+	NAIP (64.1-20.44 Ma)				
50		Paleogene	hot-warm											+ and -					
60			warm-hot								A4 (66.6 Ma); A3 (67.1 Ma); A4 (67.3 Ma)			+ and -	NAIP (64.1-20.44 Ma) Deccan (69.37-63.97) Caribbean (141.9-69.4)		KPg	+ and -	
70			transitional-hot								A3 (70 Ma)			-					
80			transitional-warm					greenhouse			A4 (88 Ma)			+	Madagascar (92-88.3 Ma) High Arctic (126.6-79.5 Ma)				
90			hot			warm								+			CT		~ radiation of angiosperms and terrestrial arthropods [4]
100			hot-warm											+	Caribbean (141.9-69.4) Madagascar (92-88.3 Ma) High Arctic (126.6-79.5 Ma) Kerguelen (127.43-89.59 Ma) Ontong Java (127.2-92.6 Ma)				
110		Cretaceous	hot-warm				greenhouse					~ 80-125 Ma		+					
120			warm-hot						5		A3 (100 Ma)			+		lots CAVs - JUR/CRET			
130			warm	greenhouse	hot					4				+ and -	Caribbean (141.9-69.4) High Arctic (126.6-79.5 Ma) Kerguelen (127.43-89.59 Ma) Ontong Java (127.2-92.6 Ma) Paraná-Etendeka (145.77-126.9 Ma)				
140	Mesozoic							icehouse						+			Tith		
150			transitional								A1 (150 Ma)			+				pos	
160			cool-cold			cool								+ and -					
170			transitional-warm								A3 (163 Ma); A1 (165 Ma); A3 (167 Ma); A1 (169 Ma)			+ and -	Karoo (189-175 Ma)				
180		Jurassic	transitional-warm										~ 180-200 Ma	+ and -	Ferrar (183.9-181.04 Ma) Karoo (189-175 Ma)		TOAE		
190			transitional								A2 (190 Ma)			+					
200			neutral-cool								A1 (200 Ma)			+ and -	CAMP (203.7-190.6 Ma)		TJ		~ beginning Marine Mesozoic Revolution (MMR, major increase marine nutrients & change ocean buffering capacity) [7][23]
210			transitional			warm	transitional	greenhouse						+ and -					
220		Triassic									A5 (210 Ma); A3 (219 Ma)			+					
230			transitional-cool						4					-	Siberian Traps (256.7-227.9 Ma) Emeishan (265.1-247.4 Ma)				
240			warm								A5 (240 Ma)			+ and -					
250			cool-cold								A3 (255 Ma); A4 (258 Ma)			+ and -			PT		
260		Permian	cool								A3 (264.7 Ma); A4 (265.3 Ma)		~ 260-280 Ma	+ and -	Emeishan (265.1-247.4 Ma) Tarim (292.2-261.7 Ma)	few CAVs - PERM	Cap (Guad)		
270			cool-cold				icehouse	icehouse			A3 (271 Ma)	~ 270-280 Ma						neg	Late Paleozoic Ice Age (LPIA ~ 330-255Ma) [6][15]
280					cold				3	3					Tarim (292.2-261.7 Ma)				
290			cool	icehouse		cold					A1 (290 Ma); A2 (299 Ma)								
300											A4 (304 Ma)			-					
310			cold																
320														-			Serp		
330														-					
340		Carboniferous	cool-cold								A5 (326 Ma)			-					
350			cool				transitional						~ 350-360 Ma	+				pos	~ beginning build-up organic matter (no decay) - major 12C burial [6]
360			transitional								A2 (360 Ma); A3 (369 Ma)			+	Yakutsk-Vilui (379.7-362.4 Ma)		Fam (DC)		
370			warm					greenhouse						+ and -			FF		
380		Devonian	transitional-warm		hot									+ and -			GIV		
390	Paleozoic		transitional			warm	greenhouse				A2 (377 Ma)							neg	
400				greenhouse					2	2								+ and -	
410			hot											+				pos	
420		Silurian	warm-hot								A2 (420 Ma)			+					
430			neutral-cool				transitional							+		few CAVs – SIL		+ and -	~ life moving onto land [11][16]
440			warm-cool	icehouse	cold	cold	icehouse	icehouse						+			ORD		
450		Ordovician	warm				transitional							+				neg	
460													~ 450-470 Ma	+					
470			hot											-					
480					hot						NO DATA			+			Tremp	pos	Great Ordovician Biodiversification Event - GOBE (upCAMB-midORD ~ 460Ma) [1][17][19]
490														+			Franc		
500				greenhouse		warm	greenhouse	greenhouse	1	1				-	Kalkarindji (511-505 Ma)		Dres		
510		Cambrian	NO DATA											+ and -		lots CAVs - CAMB			
520														+ and -			Bot	NO DATA	~ evolution predation; Cambrian Explosion [21]
530					NO DATA									+ and -					
540																			

thick horizontal lines from here = Haggis bin boundaries from column K

Judd et al 2024 [10]	Vérard 2024 [22]	Marcilly et al 2022 [13]	Scotese et al 2021 [18]	McKenzie et al 2016 [14]	Link 2009 [12]	CIEs [5]	Vulnerability (raw)
28-36 °C	greenhouse	greenhouse	20 °C	greenhouse	greenhouse	more -	bad times
	icehouse	icehouse	17.5 °C	transitional	icehouse	even +, -	good times
25-28 °C			16 °C	icehouse		more +	mixed times

References cited in compilation table.

1 Algeo, T.J., Marenco, P.J. and Saltzman, M.R., 2016. Co-evolution of oceans, climate, and the biosphere during the 'Ordovician Revolution': a review. *Palaeogeography, Palaeoclimatology, Palaeoecology*, *458*, pp.1-11.

2 Alroy, J., 2014. Accurate and precise estimates of origination and extinction rates. *Paleobiology*, *40*(3), pp.374-397.

3 Bambach, R.K., 2006. Phanerozoic biodiversity mass extinctions. *Annu. Rev. Earth Planet. Sci.*, *34*(1), pp.127-155.

4 Boyce, C.K., Brodribb, T.J., Feild, T.S. and Zwieniecki, M.A., 2009. Angiosperm leaf vein evolution was physiologically and environmentally transformative. *Proceedings of the Royal Society B: Biological Sciences*, *276*(1663), pp.1771-1776.

5 Cramer, B.D. and Jarvis, I.J.G.T.S., 2020. Carbon isotope stratigraphy. In *Geologic time scale 2020* (pp. 309-343). Elsevier.

6 Feulner, G., 2017. Formation of most of our coal brought Earth close to global glaciation. *Proceedings of the National Academy of Sciences*, *114*(43), pp.11333-11337.

7 Finnegan, S., McClain, C.M., Kosnik, M.A. and Payne, J.L., 2011. Escargots through time: an energetic comparison of marine gastropod assemblages before and after the Mesozoic Marine Revolution. *Paleobiology*, 37(2), pp.252-269.

8 Foote, M., 2023. Diversity-dependent diversification in the history of marine animals. *The American Naturalist*, *201*(5), pp.680-693.

9 Jiang, Q., Jourdan, F., Olierook, H.K. and Merle, R.E., 2023. An appraisal of the ages of Phanerozoic large igneous provinces. *Earth-Science Reviews*, *237*, p.104314.

10 Judd, E.J., Tierney, J.E., Lunt, D.J., Montañez, I.P., Huber, B.T., Wing, S.L. and Valdes, P.J., 2024. A 485-million-year history of Earth's surface temperature. *Science*, *385*(6715), p.eadk3705.

11 Lenton, T.M., Dahl, T.W., Daines, S.J., Mills, B.J., Ozaki, K., Saltzman, M.R. and Porada, P., 2016. Earliest land plants created modern levels of atmospheric oxygen. *Proceedings of the National Academy of Sciences*, *113*(35), pp.9704-9709.

12 Link, P. "Icehouse" (cold) climates. *In* Gornitz, V. ed., 2008. *Encyclopedia of paleoclimatology and ancient environments*. Springer Science & Business Media.

13 Marcilly, C.M., Torsvik, T.H. and Conrad, C.P., 2022. Global Phanerozoic sea levels from paleogeographic flooding maps. *Gondwana research*, 110, pp.128-142.

14 McKenzie, N.R., Horton, B.K., Loomis, S.E., Stockli, D.F., Planavsky, N.J. and Lee, C.T.A., 2016. Continental arc volcanism as the principal driver of icehouse-greenhouse variability. *Science*, 352(6284), pp.444-447.

15 Montañez, I.P. and Poulsen, C.J., 2013. The Late Paleozoic ice age: an evolving paradigm. *Annual Review of Earth and Planetary Sciences*, *41*, pp.629-656.

16 Otoo, B.K., 2025. Prospects for studying continentalization and the origin of terrestrial ecosystems during the late Paleozoic. *Frontiers in Ecology and Evolution*, *13*, p.1606225.

17 Rasmussen, C.M., Kröger, B., Nielsen, M.L. and Colmenar, J., 2019. Cascading trend of Early Paleozoic marine radiations paused by Late Ordovician extinctions. *Proceedings of the National Academy of Sciences*, *116*(15), pp.7207-7213.

18 Scotese, C.R., Song, H., Mills, B.J. and van der Meer, D.G., 2021. Phanerozoic paleotemperatures: The earth's changing climate during the last 540 million years. *Earth-Science Reviews*, *215*, p.103503.

19 Servais, T., Owen, A.W., Harper, D.A., Kröger, B. and Munnecke, A., 2010. The great Ordovician biodiversification event (GOBE): the palaeoecological dimension. *Palaeogeography, Palaeoclimatology, Palaeoecology*, *294*(3-4), pp.99-119.

20 Smith, F.A., Boyer, A.G., Brown, J.H., Costa, D.P., Dayan, T., Ernest, S.M., Evans, A.R., Fortelius, M., Gittleman, J.L., Hamilton, M.J. and Harding, L.E., 2010. The evolution of maximum body size of terrestrial mammals. *Science*, *330*(6008), pp.1216-1219.

21 Valentine, J.W., 2004. *On the origin of phyla*. University of Chicago Press.

22 Vérard, C., 2024. On greenhouse and icehouse climate regimes over the Phanerozoic. *Terra Nova*, 36(4), pp.292-297.

23 Vermeij, G.J., 1977. The Mesozoic marine revolution: evidence from snails, predators and grazers. *Paleobiology*, *3*(3), pp.245-258.
