# Supplementary material for: Transitions between persistent climate–carbon regimes coincide with elevated Phanerozoic biosphere vulnerability
Source: Nat Commun. 2026 Aug 4;17:7559. doi: 10.1038/s41467-026-75655-9 (PMC13439174; doi:10.1038/s41467-026-75655-9)
Supplement: Supplementary file 2 [file 41467_2026_75655_MOESM2_ESM.pdf]

nature portfolio

Peer Review File

Transitions between persistent climate-carbon regimes coincide with elevated Phanerozoic biosphere vulnerability

Corresponding Author: Dr Ivan Sudakow

This file contains all reviewer reports in order by version, followed by all author rebuttals in order by version.

Version 0:

Reviewer comments:

Reviewer #1

(Remarks to the Author)
The paper addresses a long-term issue with deep-time wiggle-matching in climate and biodiversity change studies. The issue is data heterogeneity and the risk is over-diagnosis of false positives. In other words, if two patchy records (e.g. climate proxy like oxygen, temperature, sea level vs. palaeodiversity) are compared, some model fit will be identified as most plausible, sometimes identifying a single driver, sometimes a mix of interacting drivers. There is no real solution to this dilemma, and statistical sophistication (e.g. correlation   linear model fitting   Bayesian modelling of data and error) does not resolve this dilemma of excessive type I errors.

The authors propose an entirely new approach whereby they identify packets of time when climates were more or less stable and treat these as units. They then identify times of transition between climate regimes and look at statistical ways to test whether these transitions had impacts on biodiversity or not; they find that indeed they do.

The paper is largely about mathematical approaches, and the new approach emphasizes tipping points rather than wiggle-matching, arguably a more secure approach to the question. It also recognises the close coupling of many of the drivers that earth scientists treat as if they are independent variables (e.g. oxygen, carbon, strontium, sulfur, sea level, temperature) – of course the usual practice is numerically suspect. The focus on the carbon-climate cycle fits current assumptions about ‘how the world works’. The authors provide a convincing narrative of how those transitions highlighted in their analysis correspond mainly to known times of climate perturbation associated with hyperthermal events and icehouse/ greenhouse shifts.

I think this is a very important paper that will be of wide interest, and provides a new approach to tracking Earth-Life dynamics.

Figure 1B: maybe mark with thin lines the ‘big five’ mass extinctions to help people calibrate.
Figure 4: similarly, maybe add labels to the big five: Late Ordovician, etc, etc…

Mike Benton, March 2026

Reviewer #2

(Remarks to the Author)

The manuscript "Transitions between persistent climate-carbon regimes coincide with elevated Phanerozoic biosphere vulnerability" by Sudakow et al, addresses the challenge of linking environmental changes (abiotic forcing) to biological responses (biotic responses) over the Phanerozoic eon. This is a task complicated by uncertainty, heterogeneity, and irregularity of sampling.

The authors use three complementary approaches: recurrence diagnostics to categorize Phanerozoic evolution into recurring mega-climate states, early-warning signals (EWS) to detect transitions between these states, and a climate-carbon cycle model to study long-term climate-carbon interactions. They introduce a biosphere vulnerability metric, which suggests

that high turnover coupled with low standing diversity correlates with higher vulnerability. The authors show that vulnerability increases as the system approaches regime shifts.

The paper provides a novel and comprehensive framework for understanding long-term climate–carbon dynamics and their impact on biosphere vulnerability. This is a significant contribution that also opens up potentially fruitful avenues for future research. The manuscript is clearly written, and the use of multiple methods strengthens its conclusions. I recommend it for publication in Nature Communications after minor revisions.

To ensure the paper is clearer to the broad readership of Nature Communications, I suggest the following:

- Define the Phanerozoic period when it is first introduced in the discussion, either in the Abstract or in the first line of the Introduction.

- Display the sub-periods considered in pages 4 and 5 (e.g., Cambrian–Mid Ordovician, Upper Ordovician–lowermost Carboniferous) in timeline charts showing extinction events alongside geological periods, Large Igneous Provinces (LIPs), EWS behavior and vulnerability. With relevant modifications appropriate to the current manuscript, I suggest considering something similar to Figure 4 in Ernst, R.E. & Youbi, N. (2017), Palaeogeography, Palaeoclimatology, Palaeoecology, 478, 30–52. (https://doi.org/10.1016/j.palaeo.2017.03.014)

- If some of the boundaries between geological periods do not align with the latest update of the International Chronostratigraphic Chart (ICS)-standard timelines (https://stratigraphy.org/chart/), please provide an explanation or reference. For instance, please check the timelines for the Mid Ordovician (start of page 4): instead of Cambrian–Mid Ordovician (539–447 Ma) shouldn't it be Cambrian–Late Ordovician (~541–444 Ma)?

The authors propose using biotic turnover and standardized standing diversity to develop a biosphere vulnerability metric, suggesting that high turnover combined with low diversity may indicate high vulnerability. Vulnerability typically refers to the propensity to be adversely affected, see for instance the IPCC Sixth Assessment Report (AR6), WGII Annex II: Glossary: (https://www.ipcc.ch/site/assets/uploads/2018/02/WGIIAR5-AnnexII_FINAL.pdf)
I would like them to address the following two points:

1 - the authors use two components of biotic turnover (origination rate (p) and extinction rate (q)) in their metric, treating them as playing similar roles by summing them together. However, at first glance, a high origination rate (p) suggests recovery and resilience, while a high extinction rate (q) indicates loss and increased vulnerability. In simple terms, why does biotic turnover as a whole imply vulnerability?

2 - In their use of diversity (D), do the authors consider functional diversity? While the focus is on extinction events, I wonder if functional stability might help buffer ecosystems against extinction, even during high turnover. This may be outside the scope of their main results, but it would be helpful to clarify whether their metric accounts for any aspects of this internal dynamic.

In addition to the points above, here are a few more minor suggestions to improve clarity and readability:

Optional:
Page 4, first paragraph is clear on a second reading, but I found it confusing at first. To remove ambiguity, I suggest rephrasing "This environmental regime sets the stage for…" to "The Cambrian–Mid Ordovician regime sets the stage for….." That sentence, just after the reference to Kalkarindji LIP, could be misinterpreted as implying that Kalkarindji LIP activity triggered the Cambrian Explosion, even though the explosion predates the LIP (as the paragraph says).

Page 4, first paragraph.
GOBE timing is written backwards: instead of "~460–485 Ma", it should be ~485–460 Ma

Page 4, last paragraph
Karim LIP – Tarim LIP? Check reference [35]

Figures 1A and last paragraph of Page 1: briefly explaining what "haggis" look like would make the figure easier for the general readership.

Legend of Figure 1 C and Figure 3: Define what each colour corresponds to

Reviewer #3

(Remarks to the Author)
SUMMARY RECOMMENDATION:

The manuscript presents a rigorous and sufficiently novel look at the possible interplay of Earth environment and life to warrant a wide audience and wider discussion. I was fascinated and informed by it. I recommend acceptance, subject to first asking the authors to consider possible revisions concerning clarity and necessity of their methods, especially EWS, and the necessity of the potentially irritating "haggis bin" analogy.

STRENGTHS:
This manuscript considers a familiar but fundamental question in paleobiology -- the extent of triggering of macro-evolutionary events in Phanerozoic time by environmental change. In the process, it analyzes a strong published dataset with more modern analytical tools and formalizes the appropriateness of treating the geological history of the physical environment as a small set of recurring regimes. The physical environment is represented by carbon and oxygen isotope time series as well as estimates of global mean surface air temperature and the partial pressure of carbon dioxide. The authors show that the emergent pattern of climate states is robust to the use of different proxies and statistics. It also finds support in a relatively simple climate model.

Evidence for triggering is partly and unavoidably a matter of coincident timing. Although it is often stated that "correlation is not proof of causation," the relative timing of purported cause and effect is a necessary first test. A plausible cause precedes its effect. For the paleobiological record, the authors use two different published analyses of the community Paleobiology Database (PBDB). They strengthen their results by showing the good fit between these two different numerical treatments, from two different authors.

The authors' statistical tools, Joint Recurrence Analysis (JRA) and Early Warning Signals (EWS), and their necessary pretreatment of the data are explained in the supplementary material. The authors' new vulnerability statistic is a useful addition to measures others could use to track biotic system changes through geologic time. It is helpful that they compare its formulation with the older "volatility" term. These are two ways to standardize biotic turnover relative to diversity.

POSSIBLE WEAKNESSES:
I struggled to understand Early Warning Signals (EWS) and, consequently, to convince myself that it was a necessary component of the analyses. "The appropriate time-scale separation for early warning indicators" is said by the authors to be a limitation. I guess that means it is hard to know when multiple indicators should be treated as one signal. It is troubling to learn that the JRA and EWS "require temporally equidistant sampling." This would be a harsh requirement for the graptolite example and probably much more so for such heterogeneous compilations as the PBDB.

There are rather a lot of methodological buzzwords in the early part of the paper ("tipping element thinking," "dynamical diagnostics, " "critical-transition theory," "change points" . . .). The reader struggles with some overwhelmingly abrupt changes of focus following an unfamiliar buzzword.

The second part of the paper is an impressively brief summary of the whole Phanerozoic history of Earth! A reader might emerge, however, with the sense that there are so many isotopic, volcanic, climatic and plate-tectonic events that it could be inevitable that some would appear to coincide with or immediately precede the five climate-carbon regime transitions. To some extent, the manuscript does deal with this matter of the differences in activity during transition times and during the relatively stable times of the recurrent states.

It is understandable that the data are treated at genus level. Nonetheless it is disappointing. Are species not closer to the operational level of evolutionary change? The appearance or disappearance of species that are also the first or last of a genus, might not have the same significance as species events within a genus. Genera are longer lived than species. This means that, in an incomplete record, the errors of timing of first and last occurrences are potentially larger for genera than species. It is a matter of the number of finds of the taxon within the taxon range.

A distraction and irritation:
Although familiar with life in Scotland (as a geologist, as an army officer-cadet and as a tourist), I failed to understand any analogy between mega-climate regimes and haggis, which I have eaten in both Scotland and New Zealand. Perhaps I am just an ignorant Sassenach, with no sense of humor. Fair enough. I notice, however, that the authors find it necessary to reiterate that "Haggis bin" is a stand-in for "mega climate cycle" at least six times in the manuscript and twice more in the supplementary text. They seem not to trust that "haggis bin" will be a convenient and self-evident short-hand that readers will immediately understand when it is first presented.

SUGGESTIONS:
The authors stress that both the forcing and the biotic response are heterogeneous in space and time (irregularly sampled, non-stationary, uncertain geo-proxies, uncertain dating). I would add that the local records are also inevitably stratigraphically incomplete. This is a reason that the analyses must be undertaken, as here, on interregional or global composite records. Multiple local records are necessary to fill local gaps. Gaps in the temporal record would otherwise tend to create the appearance of simultaneous change in multiple components of the record. Indeed, such a concentration of changes is an indicator of correlative hiatuses. Perhaps it would be worth emphasizing that the authors' analysis should not be applied at the local level.

The paleobiology database (PBDB), used here. is one of two community attempts to digitize the fossil record. The other, more recent, is the Nanjing-based Geobiodiversity Database (GBDB, or One-Stratigraphy). Recent publications led by Junxuan Fan or Shuzhong Shen present long Phanerozoic diversity time series. These could make a good extension of the analyses to a database built around stratigraphic sections rather than taxa and a different regional expertise among the

compilers. Of course, this is merely my wish-list for the authors' future work, not something to add to this publication.

At some future date it would be good to see the analyses extended back into the Proterozoic. Late Precambrian biodiversity has been compiled and scaled (e.g. papers by Leigh Anne Riedman)

DETAILS:
I found no omissions or disagreements between the reference list and the in-text citations.

Some items in the reference list use et. al. after the first author. I guess that this is a journal requirement when the number of authors exceeds a critical limit.

Should Haggis be capitalized in mid sentence? I do not know.

Version 1:

Reviewer comments:

Reviewer #2

(Remarks to the Author)
The authors addressed my comments and I am happy with the paper to be published.

Reviewer #3

(Remarks to the Author)
The first draft was attractive and informative, especially for the numerical methods it introduced. Some aspects of the methods were puzzling in that version. In the revised version these issues are now well resolved.

The manuscript deserves wide readership in its current form.
I value all that I have learned from your analyses. Thank you.

Minor point: Reference 35, seems to be missing critical parts.
Cramer, B.D. and Jarvis, I.J. wrote chapter 11 (Carbon Isotope Stratigraphy, pp. 309-343)
of the book "Geologic Time Scale 2020" published by Elsevier.
Comparison with citations 8, 36 and 88 suggest that the book's title and/or editors might be included.
The Elsevier editors are Gradstein, F. M., Ogg, J. G., Schmitz, M. D. and Ogg, G. M.

Open Access This Peer Review File is licensed under a Creative Commons Attribution 4.0 International License, which

permits use, sharing, adaptation, distribution and reproduction in any medium or format, as long as you give appropriate credit to the original author(s) and the source, provide a link to the Creative Commons license, and indicate if changes were made.
In cases where reviewers are anonymous, credit should be given to 'Anonymous Referee' and the source.
The images or other third party material in this Peer Review File are included in the article's Creative Commons license, unless indicated otherwise in a credit line to the material. If material is not included in the article's Creative Commons license and your intended use is not permitted by statutory regulation or exceeds the permitted use, you will need to obtain permission directly from the copyright holder.
To view a copy of this license, visit https://creativecommons.org/licenses/by/4.0/

Response to the reviews on the paper

"Transitions between persistent climate – carbon regimes coincide with elevated Phanerozoic biosphere vulnerability"

We thank the Reviewers for their constructive and valuable comments. We have carefully revised the manuscript, clearly defining key concepts and correcting minor errors. All changes are highlighted in red for easy reference. Below, we provide a point-by-point response to each comment.

REVIEWER COMMENTS

Reviewer #1 (Remarks to the Author):

The paper addresses a long-term issue with deep-time wiggle-matching in climate and biodiversity change studies. The issue is data heterogeneity and the risk is over-diagnosis of false positives. In other words, if two patchy records (e.g. climate proxy like oxygen, temperature, sea level vs. palaeodiversity) are compared, some model fit will be identified as most plausible, sometimes identifying a single driver, sometimes a mix of interacting drivers. There is no real solution to this dilemma, and statistical sophistication (e.g. correlation linear model fitting Bayesian modelling of data and error) does not resolve this dilemma of excessive type I errors.

The authors propose an entirely new approach whereby they identify packets of time when climates were more or less stable and treat these as units. They then identify times of transition between climate regimes and look at statistical ways to test whether these transitions had impacts on biodiversity or not; they find that indeed they do.

The paper is largely about mathematical approaches, and the new approach emphasizes tipping points rather than wiggle-matching, arguably a more secure approach to the question. It also recognises the close coupling of many of the drivers that earth scientists treat as if they are independent variables (e.g. oxygen, carbon, strontium, sulfur, sea level, temperature) – of course the usual practice is numerically suspect. The focus on the carbon-climate cycle fits current assumptions about 'how the world works'. The authors provide a convincing narrative of how those transitions highlighted in their analysis correspond mainly to known times of climate perturbation associated with hyperthermal events and icehouse/ greenhouse shifts.

I think this is a very important paper that will be of wide interest, and provides a new approach to tracking Earth-Life dynamics.

Figure 1B: maybe mark with thin lines the 'big five' mass extinctions to help people calibrate.

Figure 4: similarly, maybe add labels to the big five: Late Ordovician, etc, etc…

Mike Benton, March 2026

We thank the Reviewer for their kind comments. We have modified both figures as suggested.

Reviewer #2 (Remarks to the Author):

The manuscript "Transitions between persistent climate-carbon regimes coincide with elevated Phanerozoic biosphere vulnerability" by Sudakow et al, addresses the challenge of linking environmental changes (abiotic forcing) to biological responses (biotic responses) over the Phanerozoic eon. This is a task complicated by uncertainty, heterogeneity, and irregularity of sampling.

The authors use three complementary approaches: recurrence diagnostics to categorize Phanerozoic evolution into recurring mega-climate states, early-warning signals (EWS) to detect transitions between these states, and a climate-carbon cycle model to study long-term climate-carbon interactions. They introduce a biosphere vulnerability metric, which suggests that high turnover coupled with low standing diversity correlates with higher vulnerability. The authors show that vulnerability increases as the system approaches regime shifts.

The paper provides a novel and comprehensive framework for understanding long-term climate–carbon dynamics and their impact on biosphere vulnerability. This is a significant contribution that also opens up potentially fruitful avenues for future research. The manuscript is clearly written, and the use of multiple methods strengthens its conclusions. I recommend it for publication in Nature Communications after minor revisions.

To ensure the paper is clearer to the broad readership of Nature Communications, I suggest the following:

- Define the Phanerozoic period when it is first introduced in the discussion, either in the Abstract or in the first line of the Introduction.

We are grateful to the Reviewer for their constructive comments. We have revised the manuscript as suggested, adding the definition in both the Introduction, beginning at Line 29, and the Discussion, beginning at Line 255.

- Display the sub-periods considered in pages 4 and 5 (e.g., Cambrian–Mid Ordovician, Upper Ordovician–lowermost Carboniferous) in timeline charts showing extinction events alongside geological periods, Large Igneous Provinces (LIPs), EWS behavior and vulnerability. With relevant modifications appropriate to the current manuscript, I suggest considering something

similar to Figure 4 in Ernst, R.E. & Youbi, N. (2017), Palaeogeography, Palaeoclimatology, Palaeoecology, 478, 30–52. (https://doi.org/10.1016/j.palaeo.2017.03.014)

As suggested by the Reviewer, we have added a summary figure to the main text, now Figure 2; a table as suggested is already available in the Supplementary Material (see Supplementary Material S6 and Supplementary Table S2). The figure necessarily contains substantial information, and we would be happy to work with the editors and production team to ensure that the final version is presented with the best possible readability and quality. We are also happy to revise the Figure/Table further as needed by the Reviewer.

- If some of the boundaries between geological periods do not align with the latest update of the International Chronostratigraphic Chart (ICS)-standard timelines (https://stratigraphy.org/chart/), please provide an explanation or reference. For instance, please check the timelines for the Mid Ordovician (start of page 4): instead of Cambrian–Mid Ordovician (539–447 Ma) shouldn't it be Cambrian–Late Ordovician (~541–444 Ma)?

We have revised the naming of our time bins to assure matching with the most recent ICS standard timelines as suggested by the Reviewer in both the main text (Results section) and Supplemental Materials.

The authors propose using biotic turnover and standardized standing diversity to develop a biosphere vulnerability metric, suggesting that high turnover combined with low diversity may indicate high vulnerability. Vulnerability typically refers to the propensity to be adversely affected, see for instance the IPCC Sixth Assessment Report (AR6), WGII Annex II: Glossary: (https://www.ipcc.ch/site/assets/uploads/2018/02/WGIIAR5-AnnexII_FINAL.pdf)
I would like them to address the following two points:

1 - the authors use two components of biotic turnover (origination rate (p) and extinction rate (q)) in their metric, treating them as playing similar roles by summing them together. However, at first glance, a high origination rate (p) suggests recovery and resilience, while a high extinction rate (q) indicates loss and increased vulnerability. In simple terms, why does biotic turnover as a whole imply vulnerability?

We agree that origination (p) and extinction (q) do not have identical biological meanings, and we did not intend to imply that high origination is adverse in the same immediate sense as high extinction. In our metric, p+q is used as a measure of total turnover intensity, that is, the overall rate at which the standing biota is being restructured. In this interpretation, elevated origination and extinction can both indicate heightened macroevolutionary reorganization, even though extinction reflects loss whereas origination reflects replacement or recovery. Thus, vulnerability in our metric reflects high total turnover relative to standing diversity, not high origination alone. This is why we use total turnover rather than net diversification, p−q, which addresses a different

question. This interpretation is consistent with prior discussions of turnover and volatility in deep time [78,79,83].

2 - In their use of diversity (D), do the authors consider functional diversity? While the focus is on extinction events, I wonder if functional stability might help buffer ecosystems against extinction, even during high turnover. This may be outside the scope of their main results, but it would be helpful to clarify whether their metric accounts for any aspects of this internal dynamic.

We do not incorporate functional diversity into the present metric. Here "D" denotes standing generic diversity, derived from the genus-level biodiversity compilations used in this study [26,27]. We agree that functional diversity, functional redundancy, and ecological buffering are important and increasingly active topics in big data paleobiology, and that they may influence extinction vulnerability during intervals of high turnover. However, incorporating these dimensions here would require assigning functional groups consistently across the Phanerozoic and then assessing their dynamics across multiple elevated extinction intervals, which is well beyond the scope of the present study.

We have revised the manuscript to clarify this point in the Results, beginning at Line 215, and in the Methods, beginning at Line 347.

In addition to the points above, here are a few more minor suggestions to improve clarity and readability:

Optional:
Page 4, first paragraph is clear on a second reading, but I found it confusing at first. To remove ambiguity, I suggest rephrasing "This environmental regime sets the stage for…" to "The Cambrian–Mid Ordovician regime sets the stage for….." That sentence, just after the reference to Kalkarindji LIP, could be misinterpreted as implying that Kalkarindji LIP activity triggered the Cambrian Explosion, even though the explosion predates the LIP (as the paragraph says).

We thank the Reviewer for pointing this out and have modified the sentence to increase clarity as suggested (Line 92).

Page 4, first paragraph.
GOBE timing is written backwards: instead of "~460–485 Ma", it should be ~485–460 Ma

We thank the Reviewer for catching this and have fixed it as suggested (Line 95).

Page 4, last paragraph
Karim LIP – Tarim LIP? Check reference [35]

We thank the Reviewer for catching this and have fixed it as suggested (Line 129).

Figures 1A and last paragraph of Page 1: briefly explaining what "haggis" look like would make the figure easier for the general readership.

We agree that the visual analogy should be made clearer for a general readership. We have clarified the term at first use in the main text, explaining that “Haggis” bins refer to the mottled, block-like structure visible in the recurrence plots. We define the term once in the text, rather than repeating the explanation in figure captions, so that the captions can remain focused on the figure content. The relevant revision begins at Line 82.

Legend of Figure 1 C and Figure 3: Define what each colour corresponds to

We have clarified in the Figure 1 caption that the colours in Figure 1C represent an age gradient within each time window, with red indicating the oldest age and magenta the youngest. We have also made a small edit to the Figure 3 caption to state that it uses the same within-bin age-gradient colouring as Figure 1C. If the Reviewer meant something different, please let us know and we are happy to revise these figures as needed.

Reviewer #3 (Remarks to the Author):

SUMMARY RECOMMENDATION:
The manuscript presents a rigorous and sufficiently novel look at the possible interplay of Earth environment and life to warrant a wide audience and wider discussion. I was fascinated and informed by it. I recommend acceptance, subject to first asking the authors to consider possible revisions concerning clarity and necessity of their methods, especially EWS, and the necessity of the potentially irritating "haggis bin" analogy.

STRENGTHS:
This manuscript considers a familiar but fundamental question in paleobiology -- the extent of triggering of macro-evolutionary events in Phanerozoic time by environmental change. In the process, it analyzes a strong published dataset with more modern analytical tools and formalizes the appropriateness of treating the geological history of the physical environment as a small set of recurring regimes. The physical environment is represented by carbon and oxygen isotope time series as well as estimates of global mean surface air temperature and the partial pressure of carbon dioxide. The authors show that the emergent pattern of climate states is robust to the use of different proxies and statistics. It also finds support in a relatively simple climate model.

Evidence for triggering is partly and unavoidably a matter of coincident timing. Although it is often stated that "correlation is not proof of causation," the relative timing of purported cause and

effect is a necessary first test. A plausible cause precedes its effect. For the paleobiological record, the authors use two different published analyses of the community Paleobiology Database (PBDB). They strengthen their results by showing the good fit between these two different numerical treatments, from two different authors.

The authors' statistical tools, Joint Recurrence Analysis (JRA) and Early Warning Signals (EWS), and their necessary pretreatment of the data are explained in the supplementary material. The authors' new vulnerability statistic is a useful addition to measures others could use to track biotic system changes through geologic time. It is helpful that they compare its formulation with the older "volatility" term. These are two ways to standardize biotic turnover relative to diversity.

POSSIBLE WEAKNESSES:
I struggled to understand Early Warning Signals (EWS) and, consequently, to convince myself that it was a necessary component of the analyses. "The appropriate time-scale separation for early warning indicators" is said by the authors to be a limitation. I guess that means it is hard to know when multiple indicators should be treated as one signal. It is troubling to learn that the JRA and EWS "require temporally equidistant sampling." This would be a harsh requirement for the graptolite example and probably much more so for such heterogeneous compilations as the PBDB.

We thank the Reviewer for raising this important point. We agree that the role of early warning signal analysis should be stated more clearly. In the revised manuscript, we clarify that EWS is used as a complementary diagnostic of candidate transition intervals, not as the sole basis for defining the regime structure. The primary partition into long-lived climate–carbon states comes from recurrence-based analysis, while EWS is used to highlight intervals where the proxy system shows transition-like behaviour. The regime framework is therefore not dependent on EWS alone, but is supported by the convergence of recurrence diagnostics, EWS behaviour, and the conceptual climate–carbon model.

We have also clarified the preprocessing requirement. The equal-spacing step applies to the isotope proxy analyses used for recurrence diagnostics and AR(1)-based EWS estimation. The raw $\delta^{13}C$ and $\delta^{18}O$ compilations differ substantially in sampling density and age spacing, so they were placed on comparable time grids before applying the same window-based analyses to both series. This was done while keeping the same number of samples, to avoid artificial autocorrelation from oversampling. This requirement does not apply in the same way to the PBDB-derived macroevolutionary series used for the vulnerability analysis, which were handled separately as described in the Methods.

The relevant revisions begin in the Introduction at Line 64, and in the Methods at Line 322.

There are rather a lot of methodological buzzwords in the early part of the paper ("tipping element thinking," "dynamical diagnostics, " "critical-transition theory," "change points" . . .). The reader struggles with some overwhelmingly abrupt changes of focus following an unfamiliar buzzword.

We agree that some of the methodological language in the early part of the manuscript could be made clearer for a broad readership. We have therefore revised the wording to reduce unnecessary jargon while retaining the technical meaning where needed. In particular, we have replaced “tipping element thinking” with “work on Earth-system tipping elements,” “dynamical diagnostics” with “dynamical systems tools,” and revised the description of early-warning indicators so that their role is introduced more directly.

The second part of the paper is an impressively brief summary of the whole Phanerozoic history of Earth! A reader might emerge, however, with the sense that there are so many isotopic, volcanic, climatic and plate-tectonic events that it could be inevitable that some would appear to coincide with or immediately precede the five climate-carbon regime transitions. To some extent, the manuscript does deal with this matter of the differences in activity during transition times and during the relatively stable times of the recurrent states.

We agree that, across the full Phanerozoic, simply listing isotopic, volcanic, climatic, and tectonic events could make some alignments with regime boundaries seem inevitable. This is not the argument we intend to make. In our analysis, the climate–carbon regime boundaries are identified first from the proxy data, primarily through recurrence analysis. The geological and biotic events are then discussed as context for interpreting those boundaries, not as the basis for choosing them.

The manuscript also goes beyond noting temporal proximity. We compare the transition intervals with the more persistent intervals between them using the regime-stratified vulnerability analysis. The important result is that recurrence-defined climate–carbon transitions align with intervals of elevated biospheric vulnerability relative to more stable background states. This does not mean that every extinction pulse or Earth system perturbation was caused by a regime shift; rather, we interpret regime boundaries as candidate intervals when the biosphere may have been more susceptible to perturbation.

It is understandable that the data are treated at genus level. Nonetheless it is disappointing. Are species not closer to the operational level of evolutionary change? The appearance or disappearance of species that are also the first or last of a genus, might not have the same significance as species events within a genus. Genera are longer lived than species. This means that, in an incomplete record, the errors of timing of first and last occurrences are potentially larger for genera than species. It is a matter of the number of finds of the taxon within the taxon range.

We agree that species-level analyses would be valuable where sufficiently complete and consistently resolved data are available. In the present study, however, the vulnerability metric requires matched Phanerozoic-scale time series of standing diversity, origination, and extinction. The published datasets available for these quantities at this scale are genus-level datasets [26,27], so we use genus-level data consistently throughout the analysis rather than mixing taxonomic levels.

This choice is therefore mainly determined by the scale and structure of the available data. As stated in the Methods, genus-level records are generally better sampled, less sensitive to sampling and preservation biases, and more compatible with the stage-scale temporal resolution of the environmental proxy data. We agree that species-level analyses would be a valuable direction for future work in more restricted intervals or clades where comparable species-level estimates are available.

A distraction and irritation:
Although familiar with life in Scotland (as a geologist, as an army officer-cadet and as a tourist), I failed to understand any analogy between mega-climate regimes and haggis, which I have eaten in both Scotland and New Zealand. Perhaps I am just an ignorant Sassenach, with no sense of humor. Fair enough. I notice, however, that the authors find it necessary to reiterate that "Haggis bin" is a stand-in for "mega climate cycle" at least six times in the manuscript and twice more in the supplementary text. They seem not to trust that "haggis bin" will be a convenient and self-evident short-hand that readers will immediately understand when it is first presented.

We agree that the analogy should be clearer at first use, and that repeated explanations of the term are unnecessary once it has been defined. In the revised manuscript, we clarify at first use that "Haggis" bins are a descriptive shorthand for the mottled, block-like structure visible in the recurrence plots. We have also reduced repeated parenthetical explanations later in the manuscript and Supplementary Material, so that the term is defined once and then used consistently. The relevant revision begins at Line 82.

SUGGESTIONS:
The authors stress that both the forcing and the biotic response are heterogeneous in space and time (irregularly sampled, non-stationary, uncertain geo-proxies, uncertain dating). I would add that the local records are also inevitably stratigraphically incomplete. This is a reason that the analyses must be undertaken, as here, on interregional or global composite records. Multiple local records are necessary to fill local gaps. Gaps in the temporal record would otherwise tend to create the appearance of simultaneous change in multiple components of the record. Indeed, such a concentration of changes is an indicator of correlative hiatuses. Perhaps it would be worth emphasizing that the authors' analysis should not be applied at the local level.

We thank the Reviewer for this insightful and constructive recommendation. We agree that local stratigraphic records are inevitably incomplete, and that section-specific hiatuses, unconformities, or facies-controlled gaps can create an artificial concentration of apparent changes across multiple variables. This is one reason why the present framework is intended for interregional or global composite records, where combining multiple records from different settings helps mitigate the influence of local gaps, rather than for direct application to individual local sections.

We have revised the manuscript to make this boundary condition clearer. Specifically, we now state in the Introduction (Line 52) and Discussion (Line 299) that the framework is designed for composite records rather than single local sections, and we have added a short note in Supplementary Material S4 explaining why applications to individual sections would require additional treatment of section completeness, sediment accumulation, and correlation structure.

The paleobiology database (PBDB), used here. is one of two community attempts to digitize the fossil record. The other, more recent, is the Nanjing-based Geobiodiversity Database (GBDB, or One-Stratigraphy). Recent publications led by Junxuan Fan or Shuzhong Shen present long Phanerozoic diversity time series. These could make a good extension of the analyses to a database built around stratigraphic sections rather than taxa and a different regional expertise among the compilers. Of course, this is merely my wish-list for the authors' future work, not something to add to this publication.

We agree that the Geobiodiversity Database / One-Stratigraphy provides an important and complementary resource for future work, especially because of its stratigraphic-section-based structure and different compilation history. Applying the present framework to GBDB-derived Phanerozoic diversity series would be a valuable independent test of whether the regime boundaries and vulnerability patterns identified here are robust across differently constructed paleobiological databases.

For the present study, however, the vulnerability metric requires matched time series of standing diversity, origination, and extinction. We therefore use the published PBDB-derived genus-level datasets that provide these quantities in a form suitable for the present analysis [26,27]. We agree that comparison with GBDB-based reconstructions would be an interesting and important direction for future work.

At some future date it would be good to see the analyses extended back into the Proterozoic. Late Precambrian biodiversity has been compiled and scaled (e.g. papers by Leigh Anne Riedman)

We agree that extending this framework into the Proterozoic would be an interesting future direction, especially given recent efforts to compile and scale late Precambrian biodiversity records, including CONOP-based reconstructions. For the present study, however, we focus on the Phanerozoic because our analysis requires paired climate–carbon proxy structure together

with matched time series of standing diversity, origination, and extinction. Extending the approach into the Proterozoic would require a separate study, since the fossil record, dominant biological groups, stratigraphic coverage, and carbon-cycle context are not directly comparable to the Phanerozoic marine metazoan record analyzed here. We therefore agree that Proterozoic applications are valuable future work, but we have kept the present manuscript focused on the interval best suited to the current framework.

DETAILS:
I found no omissions or disagreements between the reference list and the in-text citations.

Some items in the reference list use et. al. after the first author. I guess that this is a journal requirement when the number of authors exceeds a critical limit.

Should Haggis be capitalized in mid sentence? I do not know.

We thank the Reviewer for noting this. We have made the usage consistent throughout the manuscript and will check the final capitalization against the journal's style requirements during production.

Response to the reviews on the paper

"Transitions between persistent climate – carbon regimes coincide with elevated Phanerozoic biosphere vulnerability"

We thank the reviewers for their positive assessment of the revised manuscript.

Reviewer #2
We thank Reviewer #2 for confirming that their comments have been addressed.

Reviewer #3
We thank Reviewer #3 for their thoughtful and generous comments. The remaining reference issue has been corrected. The Cramer and Jarvis reference now includes the chapter title, book title, editors, page range, publisher and year:

Cramer, B. D. & Jarvis, I. J. Carbon Isotope Stratigraphy. In *Geologic Time Scale 2020* (eds Gradstein, F. M., Ogg, J. G., Schmitz, M. D. & Ogg, G. M.) 309–343 (Elsevier, 2020).
